# Simulation modeling for stratified breast cancer screening – a systematic review of cost and quality of life assumptions

**DOI:** 10.1186/s12913-017-2766-2

**Published:** 2017-12-02

**Authors:** Matthias Arnold

**Affiliations:** 1 Munich Center of Health Sciences, LMU, Munich, Germany; 2 Institute of Health Economics and Health Care Management, Helmholtz Zentrum München, Neuherberg, Germany; 3 Institut für Gesundheitsökonomie und Management im Gesundheitswesen, Ludwig-Maximilians-Universität München, Ludwigstr. 28 RG, 5. OG, 80539 Munich, Germany

## Abstract

**Background:**

The economic evaluation of stratified breast cancer screening gains momentum, but produces also very diverse results. Systematic reviews so far focused on modeling techniques and epidemiologic assumptions. However, cost and utility parameters received only little attention. This systematic review assesses simulation models for stratified breast cancer screening based on their cost and utility parameters in each phase of breast cancer screening and care.

**Methods:**

A literature review was conducted to compare economic evaluations with simulation models of personalized breast cancer screening. Study quality was assessed using reporting guidelines. Cost and utility inputs were extracted, standardized and structured using a care delivery framework. Studies were then clustered according to their study aim and parameters were compared within the clusters.

**Results:**

Eighteen studies were identified within three study clusters. Reporting quality was very diverse in all three clusters. Only two studies in cluster 1, four studies in cluster 2 and one study in cluster 3 scored high in the quality appraisal. In addition to the quality appraisal, this review assessed if the simulation models were consistent in integrating all relevant phases of care, if utility parameters were consistent and methodological sound and if cost were compatible and consistent in the actual parameters used for screening, diagnostic work up and treatment. Of 18 studies, only three studies did not show signs of potential bias.

**Conclusion:**

This systematic review shows that a closer look into the cost and utility parameter can help to identify potential bias. Future simulation models should focus on integrating all relevant phases of care, using methodologically sound utility parameters and avoiding inconsistent cost parameters.

**Electronic supplementary material:**

The online version of this article (10.1186/s12913-017-2766-2) contains supplementary material, which is available to authorized users.

## Background

Stratified breast screening aims at improving routine screening by allowing a stratification between risk groups. Stratified screening protocols could then be developed for high-risk and low-risk groups, and the balance between harmful and beneficial screening effects could be recalibrated. Owing to the complex nature of stratified screening programs and the massive cost implications of randomized control trials, simulation modeling is often the only method available or feasible for economic evaluation. Health economic modeling aims to support political decision-making, but its results are often very diverse. Part of this diversity was found to be related to a significant diversity in simulation techniques and modeling approaches.

A recent review by Elkin et al. [1] compared simulation models for stratified cancer interventions in 2011 with the aim of evaluating the risk stratification mechanism, which they call the targeting mechanisms. They found that the targeting mechanism is rarely included in the decision analytical models, but influences the results of cost-effectiveness studies substantially. Three years later, Hatz et al. [2] provided an overview of health economic assessments of personalized medicine. The authors summarized how stratified approaches do not necessarily lead to superior or inferior cost-effectiveness compared with existing health care approaches. They also found that stratified screening was often more cost-effective than stratified treatment but, overall, the variation in these studies was too substantial to reach a conclusion. Koleva-Kolarova et al. [3] reviewed simulation models for population-based screening programs with the aim of providing recommendations for future modeling endeavors. They assessed seven original models and compared disease, population and intervention input parameters as well as modeling approach and outcomes. They found that all of them predicted mortality reduction similar to randomized control trials. However, all of them were also prone to bias, mainly due to a lack of external validation and due to “lack of systematic evaluation of evidence to calibrate the input parameters” [4]

Owing to the large variety in personalization approaches, systematic reviews struggle with comparing the specific stratification suggestions in the complex continuum of care for breast cancer. Onega et al. [5] realized that a conceptual model for the comparison of stratified screening approaches was required and suggested a framework based on the steps of care delivery in stratified screening. Their framework described the complete continuum of breast screening from risk assessment to treatment and thus supported the assessment of the care continuum in simulation models for stratified screening. A systematic review focusing on the integration of the phases of care and an assessment of the cost and utility parameters used in each of the phases thus might be helpful to further assess the simulation models and evaluate if the underlying structural assumptions are appropriate for the respective research task.

This article describes such a systematic review and presents an analysis of cost and utility parameters using the Onega framework [5]. It assesses simulation models for stratified breast cancer screening according to the integration of the phases of care delivery and illustrates the variation in cost and utility parameters. By focusing on their validity and their potential impact on results, the importance of the respective phase of care for the evaluation can be assessed and potential of bias can be identified. Its aim is not to evaluate if stratified screening is superior to routine screening, but to evaluate the economic modeling approaches in this field.

## Methods

### Identifying research evidence

Stratification can be used in many areas of the breast cancer patient pathway. Onega et al. [5] describe a framework for stratified screening for breast cancer. We used an adaptation of their framework to categorize screening approaches into clusters focusing on risk assessment, detection, diagnosis or breast cancer treatment. This study focuses on approaches aiming at the stratification of patient groups into risk levels and the selection of the best screening strategy for each risk group.

### Study selection

The systematic literature search and the study selection closely follow the guidelines of the PRISMA^1^ statement [6]. The search strategy uses very broad descriptions for stratification (or personalization), the screening for breast cancer and also for studies including cost-effectiveness. The search strategy uses MEDLINE^2^ databases (also including the MEDLINE in-process and non-indexed database), Embase database, Centre for Reviews and Dissemination (CRD) databases (providing access to DARE,^3^ NHS EED^4^ and HTA^5^ databases) and Econlit databases. Search terms included “economic evaluation”, “cost”, “benefits and harms”, “screening”, “breast cancer”, “mammography”, “magnetic resonance imaging”, “personalized”, “risk-stratified” and “targeted”. Keywords and synonyms were used in titles and abstracts. The search string for each database can be found in Additional file 1: supplementary material S1.

Since the terminology for simulation modeling is quite diverse, no specific search term was used for the database search. The search strategy thus was designed to identify economic evaluations for personalized breast cancer screening. In order to identify simulation models, all identified economic evaluation were screened for the population in their methodology. If simulated or hypothetical populations were used, studies were identified as simulation models. Studies of interest use comparative simulation approaches and compare a variety of screening strategies, of which one needs to be routine mammography screening and at least one needs to suggest a stratified screening approach. They do not necessarily need to reflect the current technology or current research, but rather a fitting economic evaluation. The literature search results are then filtered using the following inclusion criteria:

- Indication: Exclusively breast cancer
- Focus on new screening strategies, not on methods to increase participation in existing strategies.
- Study type: Economic evaluation using simulation modeling
- Evaluation approach: Comparison of risk-stratified screening vs one-size-fits all screening

Exclusion criteria further filter out non-peer-reviewed publications such as conference abstracts, commentaries or study protocols, economic evaluations with updates, economic evaluations that do not use a simulation approach or only review other simulations, economic evaluations that do not use utility values, studies focusing primarily on women with a specific socio-economic or racial background, which are not comparable to other studies. The literature search and evaluation were conducted with the help of a second researcher and a review protocol.

### Literature appraisal and data extraction

Literature appraisal is based on an overview of reporting guidelines [7] and challenges in the field of the economic evaluation of personalized medicine as formulated by Annemans et al. [8]. The overview [7] compares the most commonly used quality appraisal tools for health economic modeling [9–11]. The list extracted from this review [11] adds additional elements [8]. Annemans et al. [8] described ten challenges in the economic evaluation of personalized medicine. While some of these items are already adequately reflected in existing quality appraisal tools, such as the importance of defining the scope of the economic evaluation, others are not yet completely addressed, for example the special importance of incorporating both test and intervention specifications into the model. This quality appraisal helps to establish a benchmark for a comparison of the study quality for economic evaluations in personalized medicine. A second researcher validated the quality appraisal. Additional file 1: Supplementary material S2 includes the checklist and explanation of the new items as well as an illustration of the definition of good quality used for the quality criteria.

**Fig. 1:**
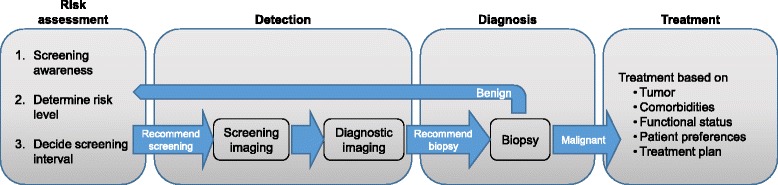
Conceptual framework, adapted from Onega et al. [5]

Data extraction utilizes the framework in Fig. 1. The framework uses four phases of care delivery in the patient’s pathway: risk assessment, screening, diagnostic work up and treatment. In each of these phases, costs can occur and quality of life can be affected. Data extraction focuses on the price parameters of technologies and quality of life decrement used in each of these phases. All monetary parameters are standardized to 2014 USD, as the latest available year of purchasing power-parity-based (PPP) exchange rates, and USD, as the most common currency. Quality of life decrements are reported as percentages from the base value in order to normalize utilities between studies using age-specific utility weights and studies assuming perfect health independent of age.

## Results

### Search results

**Fig. 2:**
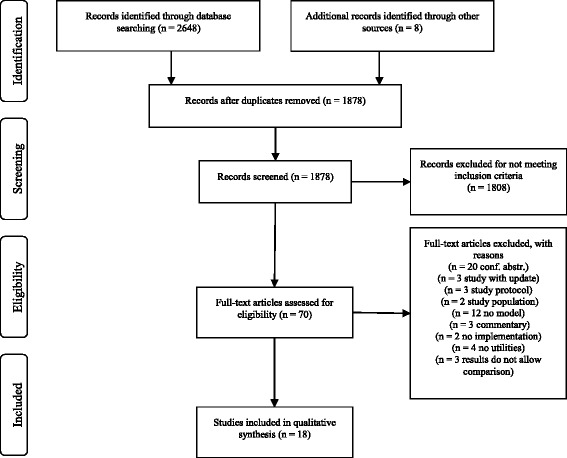
PRISMA flow chart

The search was run on 17th August 2017 and identified 2656 studies, 1251 from Embase, 944 from MEDLINE, 69 from Pre-MEDLINE, 379 from CRD and 5 from Econlit and 8 additional references per hand search. After removing duplicates, 1878 studies were assessed for inclusion criteria. Of these, 771 studies did not focus on breast cancer, 652 were not cost-effectiveness studies, 144 did not focus on screening, 107 studies did not assess personalized approaches and 134 studies focused on strategies for raising screening uptake or re-attendance. 70 studies remained and were assessed for eligibility. Of these, 52 studies were excluded because they were conference abstracts, outdated versions of newer publications, study protocols or comments on other papers, did not describe results for risk groups, focused on co-morbid study populations, did not apply health economic models, did not measure quality of life with utilities or only described models without implementing them. In all, 18 studies fulfilled all criteria and were included in this review. The PRISMA flow chart (Fig. 2) illustrates the results of the literature search [6].

### Personalization approaches

In 18 studies, three distinct clusters of stratification approaches were identified. One cluster focuses on stratified screening in the general population, one focuses on a pre-selected high-risk population and one evaluates newly introduced risk assessment technologies. Table 1 provides an overview of suggested personalization approaches, risk factors used for stratification, the routine strategy used for comparison, and effects on cost, utilities and the incremental cost-effectiveness ratio (ICER).

**Table 1:** Personalization approaches, corresponding incremental cost and utility, ICER

Cluster	Study, study country	Risk factors	Personalization approach	Proposed strategy for low/high risk group	Population and comparative strategy	Effect or utility increment	Cost increment in USD	ICER (USD per QALY)
Cluster 1: screening in general population	[12], USA	Age, breast density, family history	Screening frequency	Initial SFM at 40 years Low: 3-to-4-year interval, 50–79 years Average: biennial, 50–79 years Moderate: biennial, 40–79 years	General population, 40–79 years without screening	Not stated	Not stated	Not stated, but <$100,000 /QALY
	[16], USA	Age, breast density	Screening frequency	Low: DM, biennial, 40–79 years Moderate: DM, annual, 40–79 years	General population, 50–79 years with biennial DM	0.03% higher utility	20.8% higher cost ($730)	$151,560 /QALY
	[14], USA	Breast density	Screening technology	Low: DM, biennial, 50–79 years Moderate: DM + US, biennial, 50–79 years	General population, 50–79 years with biennial DM	0.005% higher utility	12% higher cost ($370)	$246,000 /QALY
	[17], USA	Breast density, age, other relative risks (1 to 4)	Screening frequency	Low: DM, triennial, 50–74 years, Average: DM, biennial, 50–74 years Moderate: DM, annual, 50–74 years	General population, 50–74 years with biennial DM	Not stated	Not stated	Not stated, but <$100,000/QALY
	[15], USA	Age, breast density	Screening technology	Low: SFM, annual, 40+ years Moderate: DM, annual, 40+ years	General population, 40+ years with annual SFM	0.001% higher utility	6.0% higher cost ($139)	$69,575 /QALY
	[13], USA	Age, breast density, family history (4 risk groups)	Screening frequency	Low: SFM, 4 years, 50–69 years Average: SFM, 4 years, 45–74 years Moderate: SFM, annual, 54–74 years	General population, 50–79 years with biennial SFM	3.8% higher utility	8.9% lower cost (−$124)	Dominant
Cluster 2: screening in high risk population	[19], Spain	Age, lifetime risk (>25%)	Screening technology and frequency	High: MRI / DM + CBE alternation, annual, 30–74 years	High risk population, 30–74 years with biennial MRI	0.04% higher utility	3.8% higher cost ($1379)	$59,198 /QALY
	[22], USA	Age, BRCA1/2	Screening technology	High: MRI / DM alternation, biannual, 30+ years	BRCA population, 30+ years with annual DM	0.2% higher utility	10.3% higher cost ($10,239)	$70,128 /QALY (BRCA1) $203,863/QALY (BRCA2)
	[21], USA	Age, family history (lifetime risk >15%)	Screening technology	High: MRI, annual, 25–50 years	High risk population, 25–50 years with annual SFM	0.7% higher utility	281% higher cost ($11,598)	$115,983 /QALY
	[23]	BRCA1	Screening technology	High: SFM + MRI annual, 30–49 years	BRCA population, 30–49 years, with annual SFM	0.9% higher utility in 30–39 and 1.8% in 40–49	41% higher cost in 30–39 and 34% in 40–49	$15,525 /QALY in 30–39 year olds $8987 /QALY in 40–49 year olds
	[24], USA	BRCA1	Screening technology	High: SFM + MRI, annual, 25–70 years	High risk population, 25–70 years with annual SFM	0.4% higher utility	10.6% higher cost ($9469)	$57,737 /QALY
	[25], USA	Age, BRCA1/2	Screening technology	High: MRI, annual, 25–29 years; MRI + SFM, annual, 30–49 years; SFM, annual, 50–75 years	High risk population, 25–79 years with annual SFM	0.4% higher utility	90.2% higher cost ($3484)	$38,708 /QALY
	[20], Canada	High breast density	Screening frequency	High: SFM, annual, 50–79 years	High risk population, 50–79 years with biennial SFM	0.01% higher utility	42.5% higher cost ($579)	$413,571 /QALY
	[26], Canada	Age, BRCA1/2	Screening technology	High: MRI + SFM, annual, 35–54 years	BRCA population, 25–69 years with annual SFM	1.1% higher utility	21.2% higher cost ($10,626)	$45,725 /QALY (BRCA1) $107,832 /QALY (BRCA2)
	[27], USA	Age, BRCA1/2, lifetime risk (>20%)	Screening technology	High: MRI + SFM at age 40 years	High risk population at 40 years with SFM	0.1% higher utility	34% higher cost ($589)	$21,189 /QALY
Cluster 3: screening after risk assessment	[31], USA	Gail risk classification, 7SNP	Risk assessment plus screening technology	Initial 7SNP testing Low: SFM, annual, 40–75 years High: MRI, annual, 40–75 years	General population, 40–75 years with Gail testing and the same screening strategy	0.05% higher utility	7.6% higher cost ($503)	$158,318 /QALY
	[29], USA	BRCA1/2, family history (lifetime risk >10%)	Risk assessment plus prophylactic surgery plus screening	Initial BRCA1/2 testing Low: no screening High: risk reduction surgery; MRI + SFM, annual, 30+ years	High risk population (Ashkenazi), 30+ years with family history based testing	0.1% higher utility	3.6% lower cost (−$83)	Dominant
	[28], UK	Age, high risk (5-year Gail risk >1.67%), atypia	Risk assessment plus chemoprevention plus screening	Initial atypia testing at 40 years Low: annual SFM, 40–74 years High: tamoxifen prevention, 40–74 years	High risk population, 40–74 years with annual SFM	0.5% higher effect	Higher cost^a^ (US $1357)	US $6463/QALY

SFM: screen-film mammography, DM: digital mammography, MRI: magnetic resonance imaging, CBE: clinical breast examination, BRCA1/2: breast cancer type 1/2 susceptibility protein

^a^The authors do not assess the baseline strategy; they state zero cost for mammography screening. Thus, it is impossible to provide the relative cost increase.

#### Cluster 1: Personalized screening in the general population

Studies in cluster 1 use risk factors describing moderate risk to generate risk clusters. These risk factors are for example familial risk, age, breast density, history of biopsy and others. Schousboe et al. [12] and Vilaprinyo et al. [13] use a relative risk of 1.5 for women with breast cancer history in a first-degree relative or previous biopsy and a spread of relative risk between 0.49 and 1.97 for the four categories of breast density. Sprague et al. [14] and Tosteson et al. [15] use only breast density as a risk factor. Sprague et al. [14] use the same relative risks between 0.5 and 2.0 as Schousboe et al. [12] and Vilaprinyo et al. [13]. However, Tosteson et al. [15] use only two categories of breast density with relative risks of 0.66 and 1.5; a much narrower risk spectrum. Stout et al. [16] uses only breast density, however with a scale between 1.0 and 4.35 and the necessary adjustment of lifetime risk. Trentham-Dietz et al. [17] use undefined relative risks between 1 and 4 and accordingly focus only on women with normal or increased risk, but they do not include women with relative risks below 1 as the other studies.

Sprague et al. [14] evaluate supplemental ultrasonography for women at moderate risk due to high breast density. Tosteson et al. [15] evaluate digital mammography compared to screen-film mammography for women at moderate risk; a suggestion, which is already outdated since most mammographic center are already using digital mammography in the USA today [18]. The other studies in cluster 1 suggest personalized screening frequencies. Stout et al. [16] evaluate extending screening from 50 to 40 years and increasing the screening frequency from biennial to annual for women with high breast density. Schousboe et al. [12], Vilaprinyo et al. [13] and Trentham-Dietz et al. [17], suggest triennial mammography screening for 50-year-old women with normal risk annual or biennial intervals for high-risk women at 40 or 45 years.

#### Cluster 2: Screening women at high risk

In cluster 2, studies focus on identifying the right screening technology for women already identified with high risk of breast cancer. Most studies focus on BRCA1/2^6^ positive women, only three studies [19–21] focus on other sources of high risk. Ahern et al. [19] suggest alternating magnetic resonance imaging (MRI) and mammography plus clinical breast examinations (CBE) every year instead of screening only with MRI every two years for women with lifetime risk over 25% at 25 years. Pataky, Ismail et al. [20] focus on women with pre-selected high breast density. They evaluate using annual mammography screening instead biennial for this risk group.

The other studies in cluster 2 focus on BRCA1/2 positive women. Studies suggest stratification by adding MRI for women at very high risk. Cott Chubiz et al. [22] suggest alternating MRI and mammography every 6. The other studies [23–26] propose annual screening using both technologies. Taneja et al. [27] use a single screening event instead of repeated screening.

#### Cluster 3: Additional risk assessment plus screening

In cluster 3, studies assess the introduction of additional risk assessment to stratify women according to their risk. The focus in these studies is on an earlier stage of the care continuum compared to the studies in cluster 1 and 2. Ozanne and Esserman [28] evaluate atypia testing to identify women for tamoxifen prevention. Manchanda et al. [29] evaluate BRCA gene testing compared to an assessment of family history in an Ashkenazi-Jewish population, who have a very high risk of carrying BRCA positive genes [30]. Folse et al. [31] compare the Gail tool [32] to 7SNP^7^ genetic testing to select women for routine or intensive screening.

### Quality assessment using quality appraisal checklist

**Fig. 3:**
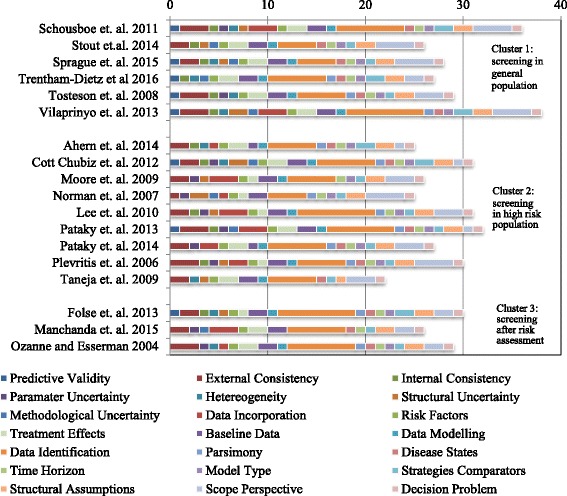
Quality appraisal, sum of positive answers

Figure 3 presents the results of the quality assessment with the help of the criteria list. When assessing the quality of simulation studies, the quality of the actual simulation can only be assessed as far as all quality-relevant items are reported in the main article, supplementary information or referenced articles and websites. In some cases, the actual quality of the simulation model might be higher, but cannot be assessed since the relevant items were not reported in the article or referenced article.

### Overall reporting quality is mixed

The criteria list includes 40 items with 40 positive answers as the maximum possible score. Longer bars in Fig. 3 indicate higher numbers of positive answers and thus high quality, whereas shorter or missing bars indicate lower quality. The bars use different colors to identify the quality categories. The complete checklist and an explanation of the additional criteria can be accessed in Additional file 1: supplementary material S2. Figure 3 shows that no article actually reaches 40: the highest scores are 38 by Vilaprinyo et al. [13] and 36 by Schousboe et al. [12]. Both studies supply extensive supplementary material describing important assumptions and calculations in their simulation and thus reach the highest transparency. The lowest scores are 22 [27] and 25 [19, 23]. All clusters have at least one study with a quality of 30 or more positive answers, but there is significant heterogeneity regarding reporting quality in all clusters.

### Personalized screening imposes challenges on decision analytic modeling

Two items should be explicitly highlighted, since they reflect the challenges of reporting stratified screening [8]. Annemans et al. [8] raised the issue that interventions of personalized medicines always consist of a combination of diagnostic and treatment with a degree of uncertainty in both technologies, which is not always adequately reflect in economic modeling. Their suggestions for good quality were translated into questions reflecting the context of stratified screening, which was described detail in the Additional file 1: supplementary material S2. Two of these questions are especially interesting, these questions are: 1) Is the strategy in focus described as a combination of risk assessment and screening technology? 2) Are all key input parameters incorporated into risk assessment and screening technology?

*Most studies do not adequately report or reflect how risk assessment and intervention are combined.*

All studies in cluster 1 and 3 explicitly mention the risk stratification and suggest screening technologies for each group. In cluster 2, risk assessment is routinely not integrated into the models. Only Plevritis et al. [26] explicitly mention the risk assessment leading into the stratified strategy. Regarding question 2), none of the studies incorporates all key input parameters. Studies in cluster 1 and 2 do not integrate risk assessment consistently. Potential utility effects of knowing to be at higher risk thus were not assessed. Screening is integrated as a cost driver, but is not consistently allowed to have quality of life effects. Especially studies in cluster 3 often exclude disutility from screening and diagnostic work up. However, while the integration of all relevant phases of care is desirable, there are reasons why certain elements might be out of the scope for the individual economic evaluation. The next paragraph discusses the scope and assumptions in each cluster in greater depth.

### Phases of care delivery

Table 2 shows the integration of the four phases of care delivery as reflected by cost and utility parameter in each specific phase. Accordingly, the gaps in the care delivery are especially interesting.

**Table 2:** Phases of care delivery and input parameters

Cluster	Study	Study perspective	Risk assessment		Detection (screening)		Diagnostic work up		Treatment	
			Cost	Utility	Cost	Utility	Cost	Utility	Cost	Utility
Screening in general population	[12]	Provider/Payer			✓ Charges		✓ Charges	✓ Assumption	✓ Charges	✓ EQ-5D S
	[16]	Provider/Payer			✓ Charges	✓ TTO expert	✓ Charges	✓ TTO expert	✓ Cost	✓ EQ-5D A
	[14]	Provider/Payer			✓ Charges	✓ TTO expert	✓ Charges	✓ TTO expert	✓ Cost	✓ EQ-5D A
	[17]	Not mentioned			✓ Charges	✓ TTO expert	✓ Charges	✓ TTO expert	✓ Cost	✓ EQ-5D A
	[15]	Societal and Provider/Payer			✓ Charges		✓ Charges		✓ Charges	✓ EQ-5D A
	[13]	Provider/Payer			✓ Cost		✓ Cost	✓ Assumption	✓ Cost	✓ EQ-5D S
Screening in high risk population	[19]	Societal			✓ Charges		✓ Charges		✓ Charges	✓ Expert VAS
	[22]	Not mentioned			✓ Charges	✓ Not described	✓ Charges	✓ Not described	✓ Cost	✓ Not described
	[21]	Provider/Payer			✓ Charges	✓ Not described	✓ Charges	✓ Not described	✓ Charges	✓ Not described
	[23]	Provider/Payer			✓ Cost		✓ Cost		✓ Cost	✓ TTO patient
	[24]	Societal			✓ Charges	✓ Assumption	✓ Charges	✓ Assumption	✓ Cost	✓ EQ-5D A
	[25]	Provider/Payer			✓ Charges	✓ Assumption	✓ Charges	✓ VAS - SG	✓ Charges	✓ SG patient
	[20]	Provider/Payer			✓ Cost	✓ Assumption	✓ Cost	✓ VAS - SG	✓ Charges	✓ SG patient
	[26]	Societal			✓ Charges	✓ Assumption	✓ Charges	✓ Assumption	✓ Charges	✓ Assumption
	[27]	Provider/Payer			✓ Charges		✓ Charges		✓ Charges	✓ Assumption
Screening after risk assessment	[31]	Not mentioned	✓ Charges		✓ Charges		✓ Charges		✓ Charges	✓ EQ-5D A
	[29]	Not mentioned	✓ Cost		✓ Charges				✓ Charges	✓ Assumption
	[28]	Not mentioned	✓ Cost						✓ Cost	✓ Mix

✓ indicates studies that included the respective phase in their cost or utility framework

EQ-5D S refers EQ-5D health utilities using an English tariff [56] in a Swedish population [49]

TTO expert describe expert interviews using a time-trade-off method to extract health utilities [36]

EQ-5D A refers EQ-5D health utilities using a tariff based on assumptions for disutility from breast cancer and a time-trade-off estimate for healthy individuals in an American population [50]

Expert VAS refers to visual analogue scale health utilities based on expert interviews [36]

VAS – SG refers to VAS health utilities in American women enrolled in mammography screening [57] which were transformed to represent standard gamble values

TTO patients refers to time-trade-off study with patients in the UK [58]

SG patient refers to standard gamble health utilities estimated in an American patient population [59]

Mix describes that the authors used a mean value of a selection of time-trade-off, standard gamble and rating scales [28]

### Disutility from risk assessment is not adequately reflected

For studies in cluster 1, risk assessment can be implemented without considerable cost implication, since all personalization suggestions utilize risk factors that usually are already available after the first screening. Most risk factors, such as family history with breast cancer, previous biopsies and age at menarche or menopause are readily collected at the first screening visit or are available through the first screening, in the case of the density of breast tissue. It is thus reasonable that risk assessment may not introduce additional cost. However, knowing to be at higher risk after risk assessment may cause distress [33] and thus may affect quality of life.

### Risk assessment is not necessarily perfect

Especially in cluster 2, these quality of life detriments may be substantial since women are at very high risk and thus anxiety and worry leading to quality of life losses are higher. Plevritis et al. [26], though not implementing it as a standard, acknowledge this effect in the assessment of BRCA positive women by testing potential utility losses after risk assessment and the effects of reassurance through negative screening in a sensitivity analysis.^8^ Excluding the risk assessment can limit the generalizability of results. The assumption underlying these studies is that at-risk women can be perfectly identified through established systems. However, genetic testing or risk assessment based on risk calculation does not always deliver perfect information [34, 35].

### Screening can affect quality of life

Most studies in clusters 1 and 2 include short-term utility loss from mammography screening. Only six studies [12, 13, 15, 19, 23, 27] do not integrate utility loss or at least test it in sensitivity analysis. Among the studies not integrating utility losses, those suggesting adjusted screening frequencies [12, 13, 19] may overestimate the utility gains from more intensive screening.

### Cost and disutility from diagnostic work up are most often included

Diagnostic work up, most importantly invasive procedures, are accompanied with temporal utility loss [36]. While mostly included, five studies [15, 19, 23, 27, 31] do not integrate these losses and thus overestimate the quality of life improvements from intensified screening. Two studies in cluster 3 do not include screening and diagnostic work up at all, despite using mammography screening to detect breast cancers [28, 29]. They assume that screening and diagnostic work up stay unaffected and thus are not integral to their evaluation.

### Data sources of cost parameters and perspectives

Table 2 also shows the data sources of cost parameters and the perspective of the economic evaluation. When cost parameters are based on national tariffs, they represent what the service provider charges from the national cost carrier for providing the health service. This is often the case in studies, which choose the perspective of national cost carriers. It might however not represent the actual resource consumption experienced at societal level. Instead of using payments, authors can use information from cost-of-illness studies, reflecting the actual cost occurred for service delivery. If used consistently, both types of information lead to consistent decision-making [37, 38], but special attention must be paid if cost parameters are mixed from both types of sources.

The three studies from the Cancer Intervention and Surveillance Modeling Network (CISNET) [14, 16, 17] use the same cost parameters. They use Medicare reimbursement charges and treatment cost estimates from an excess costing study [39]. The latter does, however, use prices from the same Medicare reimbursement catalogue, which is why they still represent the cost occurred at national payer (Medicare).

Cott Chubiz et al. [22] and Lee et al. [24] mix charges from the physician fee schedule for screening and biopsy cost estimation and add treatment expenditure from an excess costing study [40] with treatment cost for older women from a micro-costing study [41]. While both studies reference the same sources, the actual direct treatment cost are significantly different even after accounting for price inflation between the price years.

Pataky, Ismail et al. [20] combine screening and diagnostic work up cost from the screening program [42] and treatment charges from the medical services fee schedule [43]. Manchanda et al. [29] use mostly national tariffs from the National Institute for Health and Care Excellence (NICE), but in absence of a NICE unit price for genetic testing and counselling, they use cost estimates from trial data [44].

While most studies use the payer/provider perspective, five studies do not explicitly mention which perspective they chose [17, 22, 28, 29, 31]. Four studies explicitly stated that they use the societal perspective [15, 19, 24, 26]; all but one [26] adequately include cost occurring at patient level.

### Screening parameters and diagnostic work up

Table 3 presents the input parameters for screening and diagnostic work up phases. In cluster 1, screening prices are very homogenous (see Additional file 1: supplementary material S4 for details). The actual price for a lifetime of screening shows a considerable range, but the difference between lower and higher risk women is in almost all studies between US $2000 and US $2500. Vilaprinyo et al. [13] use a very different price range for Spain. For diagnostic work up, the CISNET studies [14, 16, 17] use the same cost and utility parameters.

**Table 3:** Screening and diagnostic work up cost and utility parameters

Cluster	Study	Cost of screening in 2014 USD over lifetime (in risk group)	Utility loss from screening (%)	Additional imaging		Biopsy		
				Probability of false positive	Diagnostic recall in 2014 USD	Probability of biopsy	Diagnostic biopsy in 2014 USD	Utility loss from work up
Screening in general population	[12]	Low: $812 Moderate: $3822	Not included	They combine imaging and biopsy		3.1–9.1%	$360	0–0.013 for 1 year
	[16]	Low:$ 2652 Moderate: $5304	0.6% for 1 week	n.a.	$131	n.a.	$863	10.5% for 5 weeks
	[14]	Low: $1972 Moderate: $3393	0.6% for 1 week	n.a.	$135	n.a.	$889	10.5% for 5 weeks
	[17]	Low: $1104 Average: $1656 Moderate: $3312	0.6% for 1 week	n.a.	$141.42	10.6%	$1354–$1442 depending on age	10.5% for 5 weeks
	[15]	Low: $2840 Moderate: $4480	Not included	6.3–6.8%^a^	SFM: $65 DM: $95 US: $58	6.3–6.8%^a^	FNA: $377 CNB: $290–$933 Surgical: $1402–$1700	Not included
	[13]	Lowe: $247 Average: $377 Moderate: $1248	Not included	2.8%	SFM: $34 US: $442	0.26%	$701	0.013 for 1 year
Screening in high risk population	[19]	$19,382	Not included	13.5% DM	DM: $166	2.95%	$636	Not included
	[22]	$14,060	DM: 0–10% for 1 week MRI: 0–20% for 1 week	5.59% SFM + US ^b^	DM: $125 US: $83	0.52%	CNB: $795–$1420 Surgical: $2397–$2499	0–30% for 2 weeks
	[21]	$20,550	1% for 1 year	They combine imaging and biopsy		5.4–10.8%	$503	11% for 1 year
	[23]	$5945	Not included	n.a.	MRI: $258 US: $56	n.a.	Biopsy: $276	Not included
	[24]	$31,635	SFM: 0–10% for 1 week MRI: 0–20% for 1 week	3–8%	SFM: $68 US: $53	0.3–0.8%	CNB: $662–$1302 Surgical: $1550–$1646	0–30% for 2 weeks
	[25]	$7659	0%	They combine imaging and biopsy		n.a.	$135	1.3% for 1 year
	[20]	$1276	0%	They combine imaging and biopsy		4.3–8.2%	$125	0.6% for 1 year
	[26]	$17,613	0–5% for 1 year	7% SFM + US	SFM: $64 US: $58 MRI: $649	1.6%	FNA: $382 CNB: $432–$792 Surgical: $1040–$1383	0–17% for 1 week
	[27]	$927	Not included	19% SFM + US	SFM: $64 US: $58 MRI: $649	7.2%	FNA: $382 CNB: $432–$792 Surgical: $1040	Not included
Screening after risk assessment	[31]	Test: $916 Low: $2765 High: $24,325	Not included	23% SFM + US	$251	9.6%	n.a.	Not included
	[29]	Test: $101 Low risk: $0 High: $14,800	Not included	Not included				
	[28]	Test: $276 Low: n.a. High: $24,140	Not included	Not included				

### Personalized screening women with lifetime risk between 15% and 25% costs between US $1276 and US $20,550

In cluster 2, the screening proposals show bigger variation in screening cost. The three studies focusing on women between 15% and 25% lifetime risk [19–21] propose screening strategies for US $1276 (annual mammography), US $19,382 (for annual screening with alternating MRI and mammography) or US $20,550 (for annual MRI). One study [19] does not include utility loss from screening and diagnostic work up, while the other studies include at least utility losses from diagnostic work up.

### BRCA gene carriers cost between US $7659 and US $31,635 depending on MRI cost and intensity

In cluster 2, proposals for BRCA positive women [22, 24–26] vary in lifetime screening cost between US $5945and US $31,635. One strategy with very high screening frequency but low cost [25] suggests 23 MRI screening events and 43 screening events from the age of 25 to 75 in a woman’s lifetime for US $7659. Pataky, Armstrong et al. [25] use significantly cheaper MRI cost, which explains why lifetime screening cost are comparatively low. For the other strategies, the actual prices are very similar (Additional file 1: supplementary material section S4); cost differences thus derive from the screening modality. Cott Chubiz et al. [22] suggest annual alternation of MRI and mammography from 50 years on. At the age of 70, each women thus would undergo 20 MRI and 20 mammography screenings for US $14,060. Two studies [23, 26] combine MRI and mammography every year, but limit screening to 35 to 54 years. In total, this sums up to 19 MRI and 19 mammography screenings for US $17,613. The remaining cost differences comes from slightly more expensive MRI screening (US $856 vs US $506). Norman et al. [23] suggest a very similar combined screening strategy for the UK, which has significantly cheaper screening prices, which explains also the significantly cheaper lifetime screening cost of US $5945. The most expensive strategy [24] consists of annual MRI and mammography from the age of 25 to 70, summing up to 45 MRI and 45 mammography screenings. For the diagnostic work up, three of the studies use very similar prices. Only one study [25] uses significantly lower price compared to the other studies, reflecting the price levels in the Canadian health system. The consistent use of low prices leads to more affordable screening and diagnostic work up. In the Canadian health system due to the lower screening prices compared to American health system, even very intensive MRI screening can be cost-effective.

### Additional risk assessments require more research

In cluster 3, initial risk assessment leads into risk stratification. Risk assessment costs from US $101 (for BRCA testing in Ashkenazi-Jewish women) over US $272 (for atypia testing using random fine-needle aspiration) to US $3677 (for 7SNP testing). Folse et al. [31] suggest annual MRI for high-risk women after 7SNP testing, which costs US $24,325 for 35 screenings between 30 and 70 years. In contrast, Manchanda et al. [29] estimate that 35 screening events of MRI and mammography cost only US $14,800 for high-risk women after BRCA testing. The cost difference derives from price differences in MRI screening, which is only US $318 [29] compared to US $695 [31]. Owing to the price assumptions of MRI screening, the actual screening cost in Manchanda et al. [29] are higher. Ozanne and Esserman [28] suggest tamoxifen prevention (US $24,140 for women between 40 and 70) for high risk and mammography screening for low-risk women; however, they do not report the actual cost of mammography screening. It is thus unclear if all relevant cost are included.

### False positive results mostly result in quality of life detriments, but extent varies

Screening produces false positive results, which may affect quality of life. While most studies analyze utility losses from diagnostic work up, seven studies exclude these effects and thus overestimate quality of life from screening [15, 19, 23, 27–29, 31]. Ozanne and Esserman [28] propose tamoxifen prevention as a screening replacement and underestimate potential quality of life losses associated with false positive screening results. The actual effect on quality of life varies in its extent and duration. In general, studies reflect a short-term (1 to 5 weeks) significant impact (10 to 30%) on quality of life. Over the course of a complete year, quality of life is reduced by 0.33% to 1.15%, which is also in line with the other studies using a yearly average. Only Moore et al. [21] assume a significant long-term effect of 11% over a complete year, which is higher than suggested by other studies [36, 45, 46]. Closer inspection of the health utilities however reveals that there is currently no methodologically sound set of health utilities for screening and diagnostic work up. Utilities implemented so far are either assumption-based or from expert interviews. This might explain why most studies restrain from implementing disutility from screening and diagnostic work up, despite there being some evidence that quality of life might be affected. The uncertainty of this parameter, however, is sometimes reflected in the sensitivity analyses. We did find that three studies tested disutility from screening [14, 21, 26]. Disutility from diagnostic work up was tested more frequently in cluster 1 (all but two studies [15, 17]) and cluster 2 (all but three studies [19, 23, 27]).

### Treatment parameters

Table 4 shows cost of and utility loss from treatment as well as the probabilities of treatment. The following section discusses four noteworthy differences in the assumptions utilized for the treatment phase.

**Table 4:** Parameters for direct cost of cancer treatment per stage

Cluster	Study	Is over-diagnosis assessed?	Lifetime risk in normal risk	Relative risk (risk factor)	Initial treatment cost per stage in USD			Utility loss per stage (%)			End of life cost in USD		
					In situ	Early invasive	Meta-static	In situ	Invasive	Meta-static	Other causes of death	Early invasive	Metastatic
Screening in general population	[12]	Yes	12.35%^a^	0.49–1.97 (BD) 0.9–1.5 (FH) 0.9–1.5 (Biop)	$8088	Local: $10,650; regional: $20,101	$31,096	10%	Local: 15%; regional: 25%	25%	Not used	Local: $28,824 Regional: $34,119	US $47,776
	[16]	Not mentioned	12.35%^a^	1–4.35 (BD)	$12,660	Local: $12,660; regional: 23,934	$36,964	10%	Local: 10%; regional: 25%	40%	Not used	Local: $34,265 Regional: $40,558	US $56,888
	[14]	Not mentioned	12.35%^a^	0.49–2.00 (BD)	$13,042	Local: $13,042; regional: $13,042	$28,239	10%	Local: 10%; regional: 25%	40%	Not used	Local: $ 35,300 Regional: $41,784	US $58,607
	[17]	Yes	12.35%^a^	1–4	$13,696	Local: $13,696; regional: $25,894	$39,991	10%	Local: 10%; regional: 25%	40%	Not used	Local: $ 35,070 Regional: $ 43,879	US $61,545
	[15]	Not mentioned	12.35%^a^	0.66–1.5 (BD)	$11,972	Local: $15,239; regional: $17,260	$ 0^c^	10%	Local: 10%; regional 25%	40%	Not used	Local: $16,939 Regional: $23,003	US $21,089
	[13]	Yes	5.8% by 75 years	0.49–1.97 (BD) 0.9–1.5 (FH) 0.9–1.5 (Biop)	Not included	Stage 1: $14,763; Stage 2: $21,665: Stage 3: $25,686	$42,115	Not included	Local: 10%; regional: 25%	25%	Not used	They included end of life cost in the treatment of metastatic cancers	
Screening in high risk population	[19]	Not mentioned	13%	Above 25% lifetime risk	Not included	Local: $12,661; regional: $23,937	$39,970	Not included	13–26%	n.a.	Not used	Local: $34,269 Regional: $40,564	US $56,896
	[22]	Not mentioned	65% by 70 years (BRCA)		US $8821	Local: $11,360; regional: $21,985	$15,162	10%	Local: 10%; regional: 25%	40%	$42,222	Local: $36,470 Regional: $38,326	$43,705
	[21]	Not mentioned	>15% (HIGH)		Not included	Local therapy: $11,160		^b^ $ 22,164	Not included		Breast cancer: 5% Node positive: 20% False neg. Node pos.: 34%		
	[23]	Not mentioned	41% by 50 years (BRCA1)		Not included	Without stages: $7508		Not included	Without stages: $7508		Not included		
	[24]	Not mentioned	65% by 70 years (BRCA)		US $20,585	Local: $35,073; regional: $58,165	$45,502	10%	Local: 10%; regional: 25%	40%	Not used	Local: US31,530 Regional: $31,530	$37,865
	[25]	Not mentioned	42.7% by 65 years (BRCA)		US $2481	Local: $7919; regional: $17,091	$11,324	3.5%	Local: 14%; regional: 32.5%	62%	Not used	Local: $19,329 Regional: 19,329	$19,329
	[20]	Not mentioned	26.6% from 50 to 79 years (BIRAD)		US $3116	Stage 1: $4145; stage 2: $6748; stage 3: $8274	$16,443	3.5%	Stage 1: 9%; Stage 2: 25%; Stage 3: 49%	55%	Not used	Not used / included in overall treatment cost	
	[26]	Yes	45–65% by 70 years (BRCA)		Not included	n.a.	$34,619	Not included	17%	41%	Not used		
	[27]	Not mentioned	20%	40% (BRCA)	$24,429	Local: $24,429; regional: $45,000	$34,619	n.a.	17%	41%	Not used		
Screening after risk assessment	[31]	Not mentioned	12.35%^a^	1.07–1.26 (7SNP)	$7734;	Stage 1: $13,889; Stage 2: $23,183; Stage 3: $18,449	$41,387	0%	Local: 10%; regional: 25%	40%	Not used	Stage 1: $40,229 Stage 2: $45,683 Stage 3: $51,733	$66,429
	[29]	Not mentioned	13%	4.08 (BRCA)	Not included	Without stages: $19,533		Not included	29%	35%	Not used	Terminal cancer care: $18,579	
	[28]	Not mentioned	12.35%^a^	3.0 (atypia)	$9271	Local: $13,809	$14,276	13%	32%	62%	Not used		

^a^The study is based on SEER incidence data [60], lifetime risk from 0 to 95 years

^b^The study identifies local and systemic therapy. The assumption here is that metastatic patients receive local therapy and systemic therapy

^c^The authors only use ongoing treatment cost

BD = breast density, FH = family history in first degree relative, Biop = previous biopsy, BCRA = gene mutation BCRA1 or 2, HIGH = unspecified high risk population; atypia = atypical hyperplasia found

### Studies vary in the treatment of in situ cancers

While most studies include the treatment of in situ cancers and the corresponding utility loss, six studies do not include in situ cancer treatment [13, 19, 21, 23, 26, 29]. More intensified screening, especially MRI screening, usually to a higher identification of in situ cancers [47, 48].

### Treatment costs are not consistent through the course of the disease

Almost all studies use stage-specific cost of treatment, only two studies [23, 29] do not distinguish stage-specific treatment cost, which reduces the benefit of early detection. Among the rest, four studies stand out which use lower treatment cost for metastatic disease than regional disease [15, 22, 24, 25]. Naturally, earlier diagnosis is less beneficial under this assumption. Similarly, another study uses lower end of life cost for metastatic patients than for regional cancer patients [15], which also reduces potential savings from early detection and contradicts the other studies.

While most studies do not use end of life cost for other causes of death, Cott Chubiz et al. [22] integrate these alternative end of life costs. In their study, non-breast cancer mortality is more expensive than mortality from ductal carcinoma in situ (DCIS), local or regional carcinoma. Only distant carcinoma are more expensive than dying from other causes. The assumption that women dying from DCIS is cheaper than women dying from other causes is not plausible. DCIS are by definition nonlethal; DCIS mortality thus can only consist of the cost of dying from other causes in women with DCIS. The question arises why women with DCIS are being treated differently than women without DCIS in their last life year.

### Utility parameters for treatment are based on one of two EQ-5D sets or assumptions

One of two sources are routinely being used for the health utility in cluster 1: A Swedish study [49], which uses an English time-trade-off (TTO) tariff on a Swedish population. Or an EQ-5D estimate from Stout et al. [50], which applies a tariff based partly on assumptions for breast cancer utility loss and an American (TTO) tariff [51] for healthy individuals to the Medical Expenditure Panel Survey [15, 50]. There are significant differences in these EQ-5D estimates. For example, the Swedish study [49] estimates 25% utility loss for metastatic disease, while the Stout utility set [14–16, 22, 24] uses 40% utility loss.

On one hand, there is the question if transferring the Lidgren tariff to the American setting is valid. On the other hand, the Stout utility set uses expert interviews for the disutility from screening and diagnostic work up, which certainly requires additional validation. While both EQ-5D sets have their pitfalls, they are methodological more robust than what is being used in cluster 2 and 3. Only one study in cluster 2 and cluster 3 use similar EQ-5D sets, while the other studies rely completely on assumptions, survey from very small samples, mixed sources or expert opinions.

### Treatment parameters are not routinely included in sensitivity analyses

The cost parameters for treatment show substantial variation in the studies and thus the question arises if changes in the cost parameter affect results. Ten studies check variations in the cost parameter with sensitivity analyses. However, none of those studies with sensitivity analyses report that results were sensitive to changes in treatment cost. Surprisingly, the three studies with the highest lifetime risk do not check robustness when treatment costs changed [22, 24, 26]. In these studies, in which the likelihood of breast cancer is very high, the cost of treatment could be a sensitive parameter. Screening prices are subject to sensitivity analyses in 14 studies, and seven studies find results to be sensitive to changes in screening prices, mostly referring to changes in the price of MRI screening. The simulation of Manchanda et al. [29] is the only one suggesting MRI screening, without checking if the MRI price is a sensitive parameter. Most of these nine studies also change utility losses from breast cancer; only two studies vary only the cost parameters. Only one study finds that results are sensitive to changes in utility changes [31]. Additional file 1: Supplementary Table S6 provides a full overview of the sensitivity analyses.

## Discussion

This systematic review assessed 18 simulation models for stratified approaches towards breast cancer screening. The approaches were clustered into three distinct groups. 1) A group of studies suggesting stratification of the general population, 2) a group of studies stratifying a pre-selected high-risk population and 3) a group of studies suggesting new risk assessment technologies. Quality appraisal was conducted using modified quality checklist [7]. Reporting quality was very diverse with only two studies [12, 13] in cluster 1 fulfilling 30 or more items of the 40-item quality checklist. In cluster 2 and 3, four studies [22, 24–26] and one study [31] had good quality using the same criteria of 30 items. In addition to the quality appraisal, simulation models were assessed for consistency in integrating all relevant phases of care, methodological sound utility parameters and the consistency and appropriateness of cost input parameters.

### Gaps in the phases of care

Studies often do not integrate in situ cancers into their models. Intensified screening often leads to higher detection of in situ carcinoma [52], especially if screened with MRI [47, 48]. In situ carcinoma may or may not progress to invasive cancers. Schiller-Fruhwirth et al. [53] identified the uncertainty about the biological relation between in situ and invasive cancers to be the root of the differences in modeling. However, treatment guidelines usually recommend treatment of in situ cancers [54, 55]. With increased screening, diagnosis of in situ carcinoma will increase and thus treatment of in situ cancers should be included in the economic evaluation. Simulation models thus do not capture all screening effects if in situ cancers are not integrated [13, 19, 21, 23, 26, 29].

The simulation models often do not integrate all relevant phases of care. Especially potential utility losses from screening and diagnostic work up are not routinely integrated. Only eleven studies integrate these utility losses, but nine studies do not address them [12, 13, 15, 19, 23, 27–29, 31]. This analysis has shown that there are currently no methodologically sound utility weights for screening and biopsy, but there is evidence that quality of life is affected at least in the short-term in screening and more importantly in diagnostic work up. By not including these utility decrements, the assumption of zero utility loss is automatically used, which does not reflect the underlying uncertainty and potentially biases the assessment of screening effects.

### Utility parameters are assumption-based, used out of their original context, or methodological not sound

Among the studies suggesting stratified screening for the general population, there are noteworthy differences. Two studies use EQ-5D utility estimates from a Swedish population for an American health care setting, which might raise the question if the Swedish estimates can be transferred to the American population. The utility estimates are significantly different compared to an American EQ-5D tariff based on similar methods. The lack of precise utility parameters was identified as a potential bias in many simulation studies [53]. Three of the studies [14, 16, 17] with lower scores in the quality appraisal utilized more than one simulation model. This research design produces higher robustness, since up to five simulation models evaluate the same strategy. On the downside though, the adaption of these models to the new research question is not reported in sufficient detail, which leads to lower reporting quality. However, all three studies integrate all relevant phases of care and use consistent cost and utility parameter, which is why these models produce currently the best available evidence for stratification screening in the general population. In the other clusters, only two studies [24, 31] use the American EQ-5D utility set for treatment effects on quality of life. The other studies use either sets based on assumption or on surveys from very small samples.

### Inconsistencies in cost parameters

On the side of the cost assumptions, four studies use inconsistent treatment parameters. Four studies use parameters for the treatment of metastatic disease, which is substantially cheaper than treatment of earlier cancer [15, 22, 25, 26]. Two studies do not distinguish the specific cancer stage in treatment [23, 29] and one study has lower end of life cost for more advanced cancers [15]. These sets of parameters appear implausible and contradict the intuition from the other simulation models. With these inconsistencies in the cost parameters, cost savings from earlier detection and treatment is reduced and the cost impact of screening is potentially biased.

This systematic review has some limitations. The focus on evaluations comparing stratified against routine screening lead to a very low number of studies, which does not necessarily represent the complete spectrum of stratified approaches. By using this restriction, many economic evaluations in the field of personalized screening may not have been part of this study. However, this systematic review assesses the integration of the care delivery framework. The integration is necessarily required for evaluations who compare risk-adapted screening against routine screening, but might not necessarily be required for studies focusing only on stratified strategies. An assessment based on the care delivery framework thus would not be fair judgement for these evaluations, which is why the studies in focus here are only those who compare personalized screening against routine screening.

The quality appraisal uses sum scores of positive answers. Sum-scores might create a misleading picture of the importance of each of the underlying questions, since they put equal weight on every question. This is in general not the intention of the quality checklists; however, this assumption allows getting a first overview before illustrating selected items in detail. In addition, quality appraisal can only assess methods and parameters if they are reported in the article, which might not always represent the true effort put into the simulation model.

This review compares cost parameters across countries using international purchasing power parities. Although this method eliminates currency and purchasing power differences, it does not take into account the health system related differences in national tariffs. In the process of standardizing parameters, additional calculations were required to enable comparison. Whenever approximations were calculated, it was indicated in the text or in a footnote.

While essential steps of this systematic review, such as the literature search and quality appraisal, used two researchers, a single reviewer conducted data extraction and analysis. For transparency, all extracted data are however presented in extensive Additional file 1: supplementary material.

## Conclusion

In this assessment, three research clusters were identified suggesting stratified screening for the general population, pre-selected high-risk populations and by using new risk assessment technologies. In 18 studies, potential biases were identified that might affect the generalizability of the respective simulation results. These potential biases consist of not integrating all relevant phases of care, using utility parameters, which are based on assumptions, are transferred out of their original context, are methodologically not sound, or by using incompatible or inconsistent cost parameters. Of 18 studies, only three studies did not show sign of potential bias.

By assessing cost and utility parameter in each phase of breast cancer care, additional insights into the validity of these simulation models could be gained. These insights could not be gotten with traditional checklist-based quality appraisals. This assessment has shown that a closer look into the cost and utility parameter can help to identify potential problems.

The challenges for decision analytical modeling, which derive from the increased complexity from personalized interventions and the interaction between risk assessment and surveillance, are not yet adequately met. Future health economic models need to pay close attention to the integration of all relevant phases of care. If methodological sound utility parameters are not available, sensitivity analyses need to be applied to reflect the underlying uncertainty regarding quality of life effects from screening and diagnostic work up. Cost parameters require close attention in order to avoid inconsistency or implausible sets for cost parameters.

## Additional file

Additional file 1: Supplementary material. (DOCX 234 kb)

## Abbreviations

7SNP: seven single nucleotide polymorphism
BRCA1/2: breast cancer susceptibility gene 1 or 2
CBE: clinical breast examination
CISNET: Cancer Intervention and Surveillance Modeling Network
CRD: Centre for Reviews and Dissemination
DARE: Database of Abstracts of Reviews of Effects
DCIS: ducal carcinoma in situ
DM: digital mammography
HTA: health technology assessment
ICER: incremental cost-effectiveness ratio
ISPOR: International Society for Pharmacoeconomics and Outcomes Research
MEDLINE: Medical Literature Analysis and Retrieval System
MRI: magnetic resonance Imaging
NHS EED: National Health Service Economic Evaluation Database
NICE: National Institute for Health and Care Excellence
PPP: purchasing power parity
PRISMA: Preferred Reporting Items for Systematic Reviews and Meta-Analyses
QALY: quality-adjusted life years
SFM: screen-film mammography
SG: standard gamble
TTO: time-trade-off
VAS: visual analogue scale

## Footnotes

1 Preferred Reporting Items for Systematic Reviews and Meta-Analyses (PRISMA)

2 Medical Literature Analysis and Retrieval System (MEDLINE)

3 Database of Abstracts of Reviews of Effects (DARE)

4 National Health Service Economic Evaluation Database (NHS EED)

5 Health Technology Assessment (HTA)

6 BRCA1 or BRCA2 stands for the breast cancer susceptibility gene 1 or 2.

7 7SNP stands for 7 single nucleotide polymorphism.

8 Sensitivity analyses are in the supplementary material S6.
