# Supplementary material for: Simulation modeling for stratified breast cancer screening – a systematic review of cost and quality of life assumptions
Source: BMC Health Serv Res. 2017 Dec 2;17:802. doi: 10.1186/s12913-017-2766-2 (PMC5712150; doi:10.1186/s12913-017-2766-2)
Supplement: Supplementary file 1 — Supplementary material. (DOCX 234 kb) [file 12913_2017_2766_MOESM1_ESM.docx]

Simulation modeling for stratified breast cancer screening – A systematic review of cost and quality of life assumptions

Supplementary material

Matthias Arnold^1,2*^

^1^ Munich Center of Health Sciences, LMU Munich, Germany

^2^ Institute of Health Economics and Health Care Management, Helmholtz Zentrum München, Neuherberg, Germany

* Correspondence to:

Matthias Arnold, Ludwig-Maximilians-Universität München, Institut für Gesundheitsökonomie und Management im Gesundheitswesen, Ludwigstr. 28 RG, 5. OG., 80539 Munich, Germany, Tel.:      +49-(0)89-2180 3579, Fax:     +49-(0)89-2180 1475, eMail: [arnold@bwl.lmu.de](mailto:arnold@bwl.lmu.de)

## S1 Literature search string

The literature search for Medline and Embase databases was conducted using the following syntax:

1. “Costs and Cost Analysis”/
2. cost.ab,ti.
3. "Cost-Benefit Analysis"/
4. economic evaluation.ab,ti.
5. (benefit* and harm*).ab,ti.
6. 1 or 2 or 3 or 4 or 5
7. Mass Screening/
8. “Early Detection of Cancer”/
9. “screen*”.ab,ti.
10. 7 or 8 or 9
11. Breast Neoplasm/
12. breast cancer.ti.
13. mamma* carc*.ti.
14. Mammography/
15. Mammography.ti.
16. “ultraso*”.ti.
17. Ultrasonography,Mammary/
18. Magnetic Resonance Imaging/
19. MRI.ti.
20. “magnetic resonance imag*”.ti.
21. 11 or 12 or 13 or 14 or 15 or 16 or 17 or 18 or 19 or 20
22. Individualized Medicine/
23. “individual*ed”.ab.ti.
24. risk.ab,ti.
25. “personali*ed”.ab,ti.
26. “stratif*.ab,ti.
27. “target*”.ab,ti.
28. 22 or 23 or 24 or 25 or 26 or 27
29. 6 and 10 and 21 and 28

The literature search for Econlit used the following search syntax:

1. AB cost OR TI cost
2. AB economic evaluation OR TI economic evaluation
3. AB benefit* and harm* OR TI benefit* and harm*scr
4. 1 OR 2 or 3
5. AB screen* OR TI screen*
6. TI mamma cancer or breast cancer
7. TI mammography
8. TI ultrasonography or ultrasound
9. TI magnetic resonance imaging or mri
10. 6 or 7 or 8 or 9
11. AB risk OR TI risk
12. AB individuali*ed or TI individuali*ed
13. AB personali*ed or TI personali*ed
14. AB stratif* or TI stratif*
15. AB target* or TI target*
16. 11 or 12 or 13 or 14 or 15
17. 4 AND 5 AND 10 AND 16

The search syntax for the CRD database, which utilised DARE, NHS EED and HTA databases, was the following:

1. (cost)
2. (economic evaluation)
3. (benefit* and harm*)
4. 1 or 2 or 3
5. (screen*)
6. (breast cancer)
7. (mammography)
8. (ultraso*)
9. (magnetic resonance imag*)
10. (mri)
11. 6 or 7 or 8 or 9 or 10
12. (personali*ed)
13. (stratif*)
14. (risk)
15. (target*)
16. 12 or 13 or 14 or 15
17. 4 AND 5 AND 11 AND 16

Study selection was conducted by two independent reviewers. Both reviewers followed the selection process as described and applied inclusion and exclusion criteria. Differences in results were solved in consensus.

## S2 Literature appraisal

For the analysis of the parameter selection, data and uncertainty handling, a combination of criteria lists was used [1-5] as suggested by Bennett and Manuel [6]. Compared to the complete list in Bennett and Manuel [6], the criteria were reduced in some items in order to avoid repetition and to focus the analysis on the cost rather than the outcome dimension. In addition to the general quality criteria for health economic evaluation and modelling in Bennett and Manuel [6], the criteria list here was supplemented by methodological challenges specific to the context of personalized medicine. Annemans et al. [7] formulated ten methodological issues in the assessment of personalized medicine. From these ten issues, those relevant to this specific context were used and translated into the criteria relevant for the economic context of personalized screening. The following section describes the methodological issues and the translation into criteria for the assessment of personalized screening:

### Importance of defining the scope / research question of the economic evaluation

Annemans et al. [7] describe the problem of defining a technology in focus in a situation where the technology consists of a combination of diagnostic and treatment. In personalized medicine, targeted treatment requires the use of specific diagnostics. Evaluating only the treatment without incorporating the diagnostic produces incomplete research results. For personalized screening, the situation is similar only that the focus is not primarily on the treatment decision but on the screening technology. The choice of the right detection technology requires specific risk assessments and vice versa. Thus it is important for economic evaluation of personalized screening technology to include the costs and positive and negative predictive values of the risk assessment which allows personalization. On the other hand, it is equally important for economic evaluations of risk assessment techniques to include cost and predictive value of the screening technology, which utilizes the new risk assessment. The question used in the review was: Is the strategy in focus described as a combination of risk assessment and screening technology?

### Sensitivity, specificity, false negatives and false positives, and predictive value need to be considered explicitly

Annemans et al. [7] recommend incorporating important input parameters, such as test sensitivity and specificity into decision analytical models. Additionally, the predictive value of a test is influenced by the prevalence of the test’s predicted risk. If the prevalence of a specific risk factor is very low, the predictive value of a test is low as well. For genetic testing such as testing for BRCA1/2 mutation carriers, the models for predicting mutation carriers as well as the mutation detection techniques carry the probability of error [8, 9]. These clinical and epidemiological input parameters are major determinants of the actual cost saving potential of a screening strategy and should be incorporated into the simulation model. The question used in the review was: Are all key input parameters incorporated into risk assessment and screening technology?

### Test characteristics should be translated into clinical utility

Annemans et al. [7] write that economic evaluation of new tests, such as genetic tests in breast cancer, are only relevant for patients if the genetic mutation in focus is related to targeted therapies promising better health outcomes. Testing only for the sake of collecting information does not provide clinical utility. Thus the development of new tests is often initiated or accompanied by the development new treatment options. This co-development needs to be addressed in the economic assessment and used in the evaluation of the test. In the case of personalized screening this relates to the question if risk factors are directly connected to different screening regimes. Better risk assessment has to be followed by a different choice in the screening regime which has to provide the possibility of earlier detection and better treatment options. In personalized screening, the translation into clinical utility needs changes in behaviour and decision in all four phases as described by Onega et al. [10]. This needs to be addressed in the economic model and accounted for. The question used in the review was: Is the clinical utility mentioned and reasonably explained?

### A combination of tests creates a more complex model and analysis

Annemans et al. [7] state that in cases where several tests are combined, the decision analytical model has to accommodate the individual key input parameters and the sequence of testing. When such combinations were used, the decision analytical model has to grow in complexity. For the analysis of personalized screening this was especially important when several risk factors are combined. One solution to this issue is to use combined input parameters. If combined parameters are not available, the combination of individual input parameters has to be verified from other sources, such as expert interviews. The question used in the review was: Are combined tests or risk assessment based on valid input parameters?

### A more complex analysis usually results in greater uncertainty

Annemans et al. [7] describe that in such cases where complex models are used, uncertainty needs to be addressed in a more detailed manner than in less complex models. Probabilistic sensitivity analysis might not be sufficient to represent the uncertainty. That is why probabilistic sensitivity analysis should be supported by analysis of structural uncertainty and scenario analysis. The question in the review was: Is the model complexity addressed in the analysis of uncertainty and accounted for?

The criteria checklist in Table S2 uses yes/no questions to assess the quality of the article in focus. While many questions are self-explanatory in what is a “yes” and what is a “no”, there are some more subjective questions that require further explanation how the decision between “yes” and “no” is made**.**

Table S2: Study appraisal checklist

| Item | Question | Source |
| --- | --- | --- |
| Decision problem | Is the objective of the evaluation specified and consistent with the stated decision problem? | [1, 5] |
| Scope / Perspective | Is the perspective of the model clearly stated? | [1, 3, 5] |
|  | Are the model inputs consistent with the stated perspective? | [1, 2] |
|  | Has the scope of the model been stated and justified? | [1, 4] |
|  | Is the strategy in focus described as a combination of risk assessment and screening technology? | [7] |
| Structural assumptions | Are the structural assumptions reasonable given the overall objective, perspective and scope of the model? | [1] |
|  | Is the clinical utility mentioned and reasonably explained? | [7] |
| Strategies / Comparators | Is there a clear definition of the options under evaluation? | [1, 3, 5] |
|  | Have all feasible and practical options been evaluated? | [1] |
| Model type | Is the chosen model type appropriate given the decision problem and specified causal relationship within the model? | [1, 4, 5] |
| Time horizon | Is the time horizon of the model sufficient to reflect all important differences between options? | [1, 3, 5] |
| Disease states / pathways | Do the disease states or the pathways reflect the underlying biological process of the disease in question and the impact of the intervention? | [1, 3, 5] |
| Parsimony | Is there indication that the structure of the model is as simple as possible and that any simplifications are justified? | [3] |
| Data identification | Are the data identification methods transparent and appropriate given the objectives of the model? | [1, 3, 5] |
|  | Are results reported in a way that allows the assessment of the appropriateness of each parameter input and assumption in the target settings? | [2] |
|  | Where choices have been made between data sources, are these justified appropriately? | [1, 2, 4] |
|  | Where data from different sources are pooled, is this done in a way that the uncertainty relating to their precision and possible heterogeneity is adequately reflected? | [2] |
|  | Are the data used to populate the model relevant to the target audiences and settings? | [2] |
|  | Has particular attention been paid to identifying data for the important parameters in the model? | [1] |
|  | Are all key input parameters incorporated into risk assessment and screening technology? | [7] |
|  | Are combined tests or risk assessment based on valid input parameters? | [7] |
|  | Has the quality of the data been assessed appropriately? | [1, 4] |
| Data modelling | Is the data modelling methodology based on justifiable statistical and epidemiological techniques? | [1, 3] |
| Baseline data | Is the choice of baseline data described and justified? | [1] |
|  | Are transition probabilities calculated appropriately? | [1, 3, 5] |
| Treatment effects | If relative treatment effects have been derived from trial data, have they been synthesized using appropriate techniques? | [1, 3] |
|  | Have the methods and assumptions used to extrapolate short-term results to final outcomes been documented and justified? Have alternative assumptions been explored through sensitivity analysis? | [1, 3, 5] |
| Risk factors | Has evidence supporting the modelling of risk factors as having an additive or multiplicative effect on baseline probabilities or rates of disease incidence or mortality been presented? | [3] |
| Data incorporation | Have all data incorporated into been described and referenced in sufficient detail? | [1, 3, 5] |
|  | If data have been incorporated as distributions, has the choice of distribution for each parameter been described and justified? | [1, 3] |
|  | If data have been incorporated as distributions, is it clear that second order uncertainty is reflected? | [1, 3] |
| Methodological uncertainty | Have methodological uncertainties been addressed by running alternative version of the model with different methodological assumptions? | [1] |
| Structural uncertainty | Is there evidence that structural uncertainties have been addressed via sensitivity analysis? | [1, 3] |
|  | Is the model complexity addressed in the analysis of uncertainty and accounted for? | [7] |
| Heterogeneity | Has heterogeneity been dealt with by running the model separately for different subgroups | [1, 3] |
| Parameter uncertainty | Are the methods of assessment of parameter uncertainty appropriate? | [1-5] |
| Internal consistency | Is there evidence that the mathematical logic of the model been tested thoroughly before use? | [1, 3] |
| External consistency | Are any counterintuitive results from the model explained and justified? | [1, 3, 5] |
|  | If the model has been calibrated against independent data, have any differences been explained and justified? | [1] |
|  | Have the results of the model been compared with those of previous models and any differences in results explained? | [1, 3-5] |
| Predictive Validity | Was the validity of the model tested? | [2, 4, 5] |

The criteria checklist in Table S2 uses yes/no questions to assess the quality of the article in focus. While many questions are self-explanatory in what is a “yes” and what would be a “no”, there are some more subjective questions that require further explanation how the decision between “yes” and “no” is made. As a rule of thumb, questions using adjectives as “sufficient”, “appropriate, “reasonable” or “adequate” are explained in the following section:

### Have all feasible and practical options been evaluated?

Studies suggesting new screening strategy require to accommodate variations of the initial strategy suggestion in order to illustrate if results are robust if the screening strategy is still a valid alternative if for example screening intervals are changed slightly, start or cessation ages are changed. While it is not required to include all possible alterations, at least some changes to the core strategy should be allowed to allow the assessment if there are core elements of the strategy which are absolutely essential for it to work. If there are not alternatives and also not explanation why the suggested strategies are without alternative this criteria is not fulfilled.

### Are the structural assumptions reasonable given the overall objective, perspective and scope of the model?

For studies evaluating personalized approaches of breast cancer screening, this question can easily be assessed when looking at the framework provided by Onega et al. [10]. One possible shortfall would be to exclude costs of genetic testing or other forms of risk assessment, while evaluating personalized strategies using this information in comparison with strategies not utilizing the information. Another shortfall deriving from the study design would be to use a societal perspective without considering the costs for patients undergoing cancer screening. This question basically checks for inconsistency in the study design and the research question and is answered with “yes” if none are found.

### Is the chosen model type appropriate given the decision problem and specified causal relationship within the model?

In economic evaluations, the choice of model type is basically between decision trees, Markov chain models and discrete event simulations (DES). While decision trees are the simplest model type of those three, they are usually only used for rather uncomplicated evaluations. When the situation is more complicated, because the risk of disease or infection is continuously increasing over a long time or there is a risk of repeated illness, decision trees are inappropriate [11]. The choice between Markov models and DES is not as straightforward since both modelling techniques have a very different approach of simulating. While Markov models use specific time periods to allow transitioning from one health state to the other, DES simulate a continuous flow of time and only allow transitioning to an event, when a specific threshold of risk has been accumulated. In general, DES is more flexible but also requires more specific input parameters that might be difficult to obtain [11, 12]. For the case of personalized breast cancer screening, both model types can be appropriate tools.

### Is the time horizon of the model sufficient to reflect all important differences between options?

Breast cancer screening programs usually recommend a screening cessation age between 70 years, for example in Germany [13] or UK [14], and 75 years, in USA [15] or the Netherlands [16]. The cessation age is usually connected to the peak in breast cancer incidence, which is between 65 and 75 years [17, 18]. The fact that screening cessation and the peak of breast cancer incidence are very close together suggests however, that there is a substantial number of women developing breast cancer even after 75 years. Decision analytical models thus should include all age until death of the patient. Accordingly, models with lifetime horizons reflect all important differences, while models with a horizon of only 80 years do not necessarily reflect late breast cancer incidence.

### Where data from different sources are pooled, is this done in a way that the uncertainty relating to their precision and possible heterogeneity is adequately reflected?

In many cases where more than one data source is used to identify cost parameters, it remains unclear if all sources stated exactly the same value. Heterogeneity between data sources could simply be assessed by data triangulation or at least be reflected in a sensitivity analysis. If parameters were pooled and heterogeneity has not been assessed and has also not been addressed in the sensitivity analysis, the question here is answered with “no”.

### Has the quality of the data been assessed appropriately?

Following Kopec at al. [4], the assessment of quality of data is dependent on the type of source. If parameters are based on literature, the quality of the literature needs to be assessed and the transferability of the result to context at hand needs to be assessed. Ideally meta-analyses are used, but high quality individual papers can also provide high quality data. Discrepancies with other sources should be identified and explained. When data is used from analysis of secondary sources, such as the SEER [19] database, quality assessment should follow the same standards as if it was from literature. If data is derived from expert elicitation interviews, quality can be assessed by data triangulation where data is compared with alternative sources or by independent experts. There are various methods to control assess the quality, in many cases the focus is on assessing the plausibility by comparing with other sources. If there is evidence that such a check for plausibility has been done, the question is answered with “yes”.

### Is the data modelling methodology based on justifiable statistical and epidemiological techniques?

As Weinstein et al. [3] point out, data generation in decision analytical models should follow standards in other fields. In the best case, every parameter would be based on qualitative and quantitative assessment as is the case in meta-analyses. In many cases, meta-analyses are not available, but heterogeneity should still be assessed and accounted for. This is especially important for the calculation of transition probabilities. When transition probabilities are based on simple mean calculations without considering heterogeneity in subgroups or when uncertainty of the mean calculation is not accounted for, this criterion is not fulfilled.

### Are the methods of assessment of parameter uncertainty appropriate?

Parameter uncertainty can be assessed deterministically by changing the point estimate to a specific value or probabilistically by drawing random numbers from previously defined distributions. Ideally deterministic sensitivity analyses do not focus on only one variable, but recognize that the variation of one variable is not independent of other variables. If such scenarios of interdependent variables are recognized, scenario analysis can be used to assess the robustness of result when the context of the study changes. In cases were uncertainty cannot be limited to such changes in the scenario, probabilistic sensitivity analysis (PSA) should be used to draw random scenarios and reflect uncertainty in many variables at once. If such a deterministic scenario analysis or PSA was used, the question is answered with “yes, if only deterministic univariate variation was allowed, so called one-way sensitivity, the answer is “no”.

## S3 Model Structure

Decision analytical modelling can be very diverse and can take many shapes and forms. Brennan et al. [20] describe a detailed taxonomy for the types of models used in health economic evaluations. They basically differentiate between cohort and patient-level simulation, between discrete and continuous time models and between models having Markovian features and models remembering the transition path for the projection of future events. In the case of personalized breast cancer screening, the decision analytical models are very diverse as Table 3 suggests, but they can be categorized into one of four groups.

### Model types

1) They can be of decision tree type, meaning they simulate cohorts of patients with a set of shared characteristics by using age-independent (untimed) transition probabilities. Brennan et al. [20] identify patient-level decision trees, where each patient is simulated with a specific probability of events occurring.

2) They can be cohort-level Markov models with a deterministic or stochastic approach to transition probabilities. The latter is often integrated with Monte Carlo simulations to introduce randomness into the transition calculation.. A Brennan, SE Chick and R Davies [20] additionally differentiate between cohort models allowing interactions and cohort models not allowing interactions. Interactions are often used when infectious diseases at focus and transition probabilities are non-independent. In the case of breast cancer screening, interactions are not commonly included and also do not appear among the studies here.

3) They can be patient-level Markov models. These kinds of models do not simulate complete cohorts, but use the individual patient as the modelling agent. They usually use Micro-Monte-Carlo simulations to generate variance in patient characteristics. Brennan et al. [20] differentiate between discrete and continuous time Markov models, whereas the latter do not use cycles of specific length but calculate the time to the next event. All these Markov models have in common that they do not allow interactions between events or individuals. There are however alternatives allowing interactions, while maintaining the Markov assumptions. One example is a discrete-time individual event history model [20].

4) Patient-level models do not necessarily need to be of Markovian type. There are models without Markovian features, most of them can be described as DES. DES-type models benefit from allowing the modelling of interaction without being restricted by the Markovian assumptions. Other than the assumption of predicting future events independently of past events, Brennan et al. [20] also highlight that DES allow using of non-Markovian distributions in stochastic models and thus are more flexible. Similar to Markov-type patient-level models, DES can use discrete and continuous time. The only difference is that DES are by nature continuous and reduce complexity to allow discrete time steps, while Markov models are by nature discrete and approximate continuous time by reducing the cycle length.

Table 1: Model types after Brennan et al. [20]

| Cluster | Study | Cohort-level models | | Patient-level models | |
| --- | --- | --- | --- | --- | --- |
|  |  | **Decision tree** | **Markovian** | **Markovian** | **DES** |
| Cluster 1: Screening in general population | **[21]** |  |  | Simulated patient-level, discrete time |  |
|  | **[22]** | Variety of models | | | |
|  | **[23]** | Variety of models | | | |
|  | **[24]** | Variety of models | | | |
|  | **[25]** |  |  |  | Non-Markovian discrete event, discrete time |
|  | **[26]** |  |  | Discrete-time individual event history |  |
| Cluster 2: Screening  in high risk population | **[27]** |  |  | Simulated patient-level, discrete time |  |
|  | **[28]** |  | Simulated cohort-level, deterministic |  |  |
|  | **[29]** |  |  | Simulated patient-level, discrete time |  |
|  | **[30]** |  | Simulated cohort-level, deterministic |  |  |
|  | **[31]** |  | Simulated cohort-level, deterministic |  |  |
|  | **[32]** |  | Simulated cohort-level, deterministic |  |  |
|  | **[33]** |  |  |  |  |
|  | **[34]** |  |  | Simulated patient-level, continuous time |  |
|  | **[35]** | Decision tree |  |  |  |
| Cluster 3: Screening after risk assessment | **[36]** |  |  |  | Non-Markovian discrete event, continuous time |
|  | **[37]** | Decision tree |  |  |  |
|  | **[38]** |  | Simulated cohort-level, deterministic |  |  |

### Modelling of tumor progression

An additional aspect of differentiation in economic modelling is the modelling of the tumor progression. Tumor progression can be described using a very simple staging; for example the progression from healthy, cancer-free women to women with a palpable mass in the breast followed by the progression to death by breast cancer. In those simple staging models, variations in tumor progression, like cases of non-progression cannot be modelled explicitly.

Many studies use summary stages to allow more variation in tumor progression. The staging most often refers to the location of the tumor, like the summary staging of the Surveillance, Epidemiology, and End Result Program (SEER) which uses four stages: In situ, localized, regional, distant [39]. In situ describes the “dormant” cancer or non-invasive tumors. Since the introduction of mammography screening, the rate of identified in situ cancers has increased dramatically. One explanation is screening often identifies dense tissue as an in situ carcinoma, which in reality is rather a lesion. Because of this misidentification, some economic models do not use these in situ carcinoma as pre-invasive cancers, which given enough time would become invasive, but rather as tissue which may or may not develop into invasive cancers. In order to reflect this uncertainty, these screening-detected in situ carcinoma are often treated as risk factors for invasive cancers rather than pre-invasive forms [21].

Once the cell wall has been breached, the tumor progresses from non-invasive to invasive and is then called a local or localized cancer in the first phase. If the tumor progresses further and extends beyond the original organ, for example when cancer cells can be found in the lymph nodes, the tumor is called regional. If the tumor spreads further, and metastases to secondary tumors in other parts of the body, the tumor is called distant [39].

Some studies also use more detailed radiographic staging systems, like the American Joint Committee on Cancer (AJCC) staging system [40]. These staging systems provide both summary stages, from stage 0 to stage 4, as well as detailed information of tumor characteristics. These characteristics are based on the tumor-node-metastasis (TNM) categories. This system measures the primary tumor size on a scale from T0 to T4, the number of affected lymph nodes from N0 to N3 and the existence or non-existence of metastases, M0 or M1. Combinations of the TNM stages are summarized into cancer stages from stage 0 for in situ cancers or T_is_N_0_M_0_ to stage 4 for metastatic cancers T_any_N_any_M_1_. One study use the TNM description [31], while most only use the summary stages of the AJCC system.

## S4 Prices of screening technologies

Overall, the cost variation in the routine screening is very low. The variation of the base case cost thus cannot be explained by the price alone. The price variation naturally is only one side of the calculation, since the frequency of screening as well as start and cessation ages need to be addressed. For the calculation in Table 3, the cost of screening is calculated by multiplying the price of the corresponding screening technology with the proposed screening frequency over the screening life span. The individual screening prices are displayed in Table S4. If authors did not provide a specific cessation age, but stated lifelong screening, cessation age of 80 years was assumed for the calculation. Diagnostic work up is presented as prices per procedure.

Table S4: Price of screening technology in 2014 USD

| **Cluster** | **Study** | **Study country** | **Price of Screening Technology in 2014 USD** | | | | |
| --- | --- | --- | --- | --- | --- | --- | --- |
|  |  |  | SFM | DM | MRI | CBE | US |
| Screening in general population | **[21]** | USA | $98 |  |  |  |  |
|  | **[22]** | USA | $79 | $136 |  |  |  |
|  | **[23]** | USA |  | $136 |  |  | $98 |
|  | **[24]** | USA |  | $138 |  |  |  |
|  | **[25]** | USA | $71 | $112 |  |  |  |
|  | **[26]** | Spain | $52 |  |  |  |  |
| Screening in high risk population | **[27]** | USA |  | $138 | $706 | $37 |  |
|  | **[28]** | USA |  | $133 | $570 |  |  |
|  | **[41]** | UK | $39 |  | $259 |  | $56 |
|  | **[29]** | USA | $72 |  | $506 |  |  |
|  | **[31]** | USA | $42 |  | $822 |  |  |
|  | **[32]** | Canada | $69 |  | $201 |  |  |
|  | **[33]** | Canada | $44 |  |  |  |  |
|  | **[34]** | USA | $71 |  | $856 |  |  |
|  | **[35]** | USA | $71 |  | $856 |  |  |
| Screening after  risk assessment | **[36]** | USA | $79 |  | $695 | $164 |  |
|  | **[37]** | UK | $52 |  | $318 |  |  |
|  | **[38]** | USA |  |  |  |  |  |

## S5 Utility base case parameters

The utility structure has shown how diverse the studies are, but there are similarities within the three clusters where utility weights are used to decrement quality of life. In order to analyse how these decrements, influence the overall quality of life, Figure 4 compares the base line utility for health individuals without breast cancer and at perfect health, for the purpose of these evaluations. These individuals might actually have other diseases or problems, but they do not have any breast cancer-related issues. The economic evaluations using quality-adjusted life years (QALY) handle this question if they are really at perfect health or they have other age-related health detriments by assigning them a utility weight of one or by using an age-specific utility weight that decreases with age. Figure 4 shows the utility weights by age and illustrates that seven studies assume their populations to stay at perfect health until death if they do not encounter breast cancer-related health problems. Eight studies assume that perfect health actually decreases over age due to age-related health problems that are not breast cancer. It has to be noted that even among those eight studies, there are different weights assigned to old age. These eight studies use QALY weights from four different sources. Two studies [34, 35] use a QALY tariff [42] based on time-trade-off assessment of women with and without chronic conditions. These tariffs are higher than all the other tariffs. Two studies [21, 26] use a Swedish tariff [43], which is based on an EQ-5D assessment. Three other studies [22, 23, 25] use a similar EQ-5D tariff, but from an assessment in a US population [44]. The lowest tariff, used by two studies [28, 29], is based on another EQ-5D tariff from an older assessment of the US population [45].

Figure 1: Age-specific utility weights in state “healthy”

## S6 Sensitivity Analysis

Best practice for simulation models is to allow variation of important input parameters. Especially, when uncertainty about their true nature or future changes might be expected, sensitivity analyses are required to find out if a variation in the variable changes the model’s result. Table S7 gives an overview of the sensitivity analysis and their impact on the model’s result as analysed in the studies.

Table S7: Sensitivity Analyses

| **Cluster** | **Study** | **Risk assessment** | | **Detection (Screening)** | | **Diagnostic work up** | | **Treatment** | |
| --- | --- | --- | --- | --- | --- | --- | --- | --- | --- |
|  |  | **Cost** | **Utility** | **Cost** | **Utility** | **Cost** | **Utility** | **Cost** | **Utility** |
| Screening in general population | **[21]** |  |  | ✓ |  |  | ✓ | ✓ | ✓ |
|  | **[22]** |  |  | ✓ |  |  | ✓+ |  |  |
|  | **[23]** |  |  | ✓ | ✓ |  | ✓ |  |  |
|  | **[24]** |  |  |  |  |  |  |  |  |
|  | **[25]** |  |  | ✓+ |  |  |  |  |  |
|  | **[26]** |  |  |  |  |  | ✓ | ✓ |  |
| Screening in high risk population | **CH Ahern, YCT Shih, W Dong, G Parmigiani and Y Shen [27]** |  |  | ✓ |  |  |  |  |  |
|  | **[28]** |  |  | ✓+ |  | ✓ | ✓ |  |  |
|  | **[41]** |  |  | ✓ |  | ✓ |  | ✓ | ✓ |
|  | **[29]** |  |  | ✓ |  | ✓ | ✓ |  | ✓ |
|  | **[31]** |  |  | ✓ | ✓ | ✓ | ✓ | ✓ | ✓ |
|  | **[32]** |  |  | ✓+ |  | ✓ | ✓ | ✓ | ✓ |
|  | **[33]** |  |  | ✓+ |  | ✓ | ✓+ | ✓ | ✓ |
|  | **[34]** |  | ✓+ | ✓+ | ✓ | ✓ | ✓ |  | ✓ |
|  | **[35]** |  |  | ✓+ |  |  |  | ✓ |  |
| Screening after  risk assessment | **[36]** | ✓+ |  | ✓+ |  | ✓ |  | ✓ | ✓+ |
|  | **[37]** | ✓ |  |  |  | ✓ |  | ✓ | ✓ |
|  | **[38]** | ✓ |  |  |  |  |  | ✓ | ✓ |
| ✓ indicates that a sensitivity analysis was conducted.  + indicates that the variable was identified to change the model’s result. | | | | | | | | |  |

Schousboe et al. [21] performed univariate and probabilistic sensitivity analysis for changing prices of screening and treatment and also utility changes from cancer treatment. However, they compared only the variation in routine screening strategies. They did not report that their assessment is sensitivity to any special variation, but it remains unclear if personalization is robust to changes. The sensitivity of the routine screening was relatively high.

Stout et al. [22] used deterministic univariate sensitivity analyses and compared their results across the five models of the Cancer Intervention and Surveillance Modeling Network (CISNET) if digital screening would drop from $139.98 to $81.35. They also allowed small utility decrements from participation in screening in the sensitivity analysis. Three of the models were very sensitive to utility changes, leading to the personalized strategy to be dominated. Cost variation changed the results quantitatively, but not qualitatively.

Sprague et al. [23] performed univariate sensitivity analyses for the cost of ultrasonography and quality losses from screening and false positive results. They reported that results were robust for all variations in the three CISNET models [22-24].

Tosteson et al. [25] performed univariate sensitivity for a decrease in the price of digital mammography. They reported that results are sensitivity to price changes. With decreasing cost, the value of targeted strategies increases and cost-effectiveness also increased. However, the results only changed qualitatively if the price of digital mammography went down to the same value of analogy mammography.

Vilaprinyo et al. [26] used univariate variations for changes in overdiagnosis, breast cancer treatment cost and disutility from false positive results. They stated that some strategies were not robust, but they did not specify which strategies. They found that changes in overdiagnosis and disutility changed the result only quantitatively, but not qualitatively.

Ahern et al. [27] changed the price of MRI in a univariate sensitivity analysis. Changes by 50% to 70% did not change the result qualitatively. However, when lifetime risk was increased by 50% and the price of magnetic resonance imaging (MRI) was reduced by 70%, the recommendation changed.

Cott Chubiz et al. [28] used univariate and multivariate deterministic analyses for changes of screening cost, diagnostic work up cost and when small utility decrements from false positive results would be allowed. Variations in the cost of MRI changed the result. If MRI cost was at 50% of the base value, the alternation strategy could be recommended, otherwise not. Disutility from false positive screening, did not change the results.

Lee et al. [29] varied cost of screening and diagnostic work up and disutility from diagnostic work up and breast cancer in univariate analyses. Especially MRI cost changes changed the recommendation towards annual combined screening strategies. Quality of life changes did not influence the model’s qualitative result.

Moore et al. [31] use univariate and probabilistic sensitivity analysis and change all cost and utility parameters. They reported that MRI could be recommended if the price dropped below $315. Variations in the other cost and utility parameter had only limited impact. The probabilistic analysis showed however, that MRI produced positive health effects in 56% of all cases with 22% producing ICERs above $100.000. Accordingly, they could not recommend the MRI strategy.

Pataky, Armstrong et al. [32] used univariate and probabilistic sensitivity analyses. They varied all cost parameters and disutility from diagnostic work up and treatment. Only cost of MRI was found to change the result qualitatively. However, in the probabilistic analysis they found that robustness was only in 56% of all parameter combinations at a threshold of $50.000/QALY. At $100.000/QALY, results were robust in 85.6% of all cases.

Pataky, Ismail et al. [33] also used univariate and probabilistic analyses. They varied all cost parameters and utility parameters for diagnostic work up and treatment. Univariate analyses showed that the cost of mammography screening can influence the result, but not cost variation of diagnostic work up or treatment. Disutility from diagnostic work up was found to change the result qualitatively. The probabilistic analysis showed that results are very sensitive disregarding the willingness-to-pay threshold.

Plevritis et al. [34] used univariate and probabilistic analysis. They changed parameters for cost of screening and diagnostic work up. In addition, they also allowed utility changes from knowing to be BRCA positive, screening, diagnostic work up and treatment. Cost of MRI were found to change the result qualitatively, and also utility changes from knowing to be BRCA positive.

Taneja et al. [35] used univariate sensitivity analyses for cost of MRI and breast cancer treatment. While MRI cost variation changed model results qualitatively, breast cancer treatment did not have qualitative impact.

Folse et al. [36] varied cost of genetic testing, screening technologies, diagnostic tests and breast cancer treatment. They also allowed changed in breast cancer treatment disutility. They found that their result is sensitive to changes in the cost of the 7SNP test, MRI screening and breast cancer disutility. The other parameters were found to only change the result quantitatively.

Manchanda et al. [37] used univariate and probabilistic sensitivity analyses for cost of genetic testing, diagnosis and treatment. They also allowed variation of breast cancer disabilities. Univariate analyses found that none of the parameters changes the result in a meaningful way and probabilistic analysis confirmed that the results are robust.

Ozanne and Esserman [38] changed cost of risk assessment and treatment as well as disutility from breast cancer in univariate analyses. Thought they do not report many detail, they state that their results are robust.

## S7 Summary of consistency issues

Table 2: Summary of results for consistency issues

| **Cluster** | **Study** | **Quality appraisal (> 30 pos answers)** | **Phases of care issues** | **Inconsistent data sources** | **Issues with screening or diagnostic work up** | **Issues with treatment** |
| --- | --- | --- | --- | --- | --- | --- |
| **Cluster 1: screening in general population** | **[21]** | pos | X |  |  | X |
|  | **[22]** | neg |  |  |  |  |
|  | **[23]** | neg |  |  |  |  |
|  | **[24]** | neg |  |  |  |  |
|  | **[25]** | neg | X |  | X |  |
|  | **[26]** | pos | X |  |  | X |
| **Cluster 2: screening in high risk population** | **[27]** | neg | X |  | X | X |
|  | **[28]** | pos |  | X |  | X |
|  | **[29]** | pos |  | X | X | X |
|  | **[31]** | neg |  |  | X | X |
|  | **[32]** | pos |  |  | X |  |
|  | **[33]** | neg |  | X |  |  |
|  | **[34]** | pos |  |  |  | X |
|  | **[35]** | neg | X |  | X |  |
| **Cluster 3: screening after**  **risk assessment** | **[36]** | pos | X |  | X |  |
|  | **[37]** | neg | X |  | X | X |
|  | **[38]** | neg | X |  | X |  |

## References

1. Philips Z, Bojke L, Sculpher MJ, Claxton KP, Golder S: **Good practice guidelines for decision-analytic modelling in health technology assessment: a review and consolidation of quality assessment**. *PharmacoEconomics* 2006, **24**(4):355.

2. Drummond MF, Sculpher MJ, Torrance GW, O'Brien BJ, Stoddard GL: **Methods for the Economic Evaluation of Health Care Programmes**, 3rd edn. Oxford: Oxford University Press; 2005.

3. Weinstein MC, O'Brien B, Hornberger J, Jackson J, Johannesson M, McCabe C, Luce BR, Studies ITFoGRP--M: **Principles of good practice for decision analytic modeling in health-care evaluation: report of the ISPOR Task Force on Good Research Practices--Modeling Studies**. In: *JVAL.* vol. 6: Elsevier Inc.; 2003: 9-17.

4. Kopec JA, Fines P, Manuel DG, Buckeridge DL, Flanagan WM, Oderkirk J, Abrahamowicz M, Harper S, Sharif B, Okhmatovskaia A *et al*: **Validation of population-based disease simulation models: a review of concepts and methods**. *BMC public health* 2010, **10**:710.

5. Nuijten MJ, Pronk MH, Brorens MJ, Hekster YA, Lockefeer JH, de Smet PA, Bonsel G, van der Kuy A: **Reporting format for economic evaluation. Part II: Focus on modelling studies**. *PharmacoEconomics* 1998, **14**(3):259-268.

6. Bennett C, Manuel DG: **Reporting guidelines for modelling studies**. *BMC medical research methodology* 2012, **12**:168.

7. Annemans L, Redekop K, Payne K: **Current Methodological Issues in the Economic Assessment of Personalized Medicine**. *JVAL* 2013, **16**(Supplement):S20-S26.

8. Antoniou AC, Hardy R, Walker L, Evans DG, Shenton A, Eeles R, Shanley S, Pichert G, Izatt L, Rose S *et al*: **Predicting the likelihood of carrying a BRCA1 or BRCA2 mutation: validation of BOADICEA, BRCAPRO, IBIS, Myriad and the Manchester scoring system using data from UK genetics clinics**. *J Med Genet* 2008, **45**(7):425-431.

9. Fischer C, Kuchenbacker K, Engel C, Zachariae S, Rhiem K, Meindl A, Rahner N, Dikow N, Plendl H, Debatin I *et al*: **Evaluating the performance of the breast cancer genetic risk models BOADICEA, IBIS, BRCAPRO and Claus for predicting BRCA1/2 mutation carrier probabilities: a study based on 7352 families from the German Hereditary Breast and Ovarian Cancer Consortium**. *J Med Genet* 2013, **50**(6):360-367.

10. Onega T, Beaber EF, Sprague BL, Barlow WE, Haas JS, Tosteson AN, M DS, Armstrong K, Schapira MM, Geller B *et al*: **Breast cancer screening in an era of personalized regimens: a conceptual model and National Cancer Institute initiative for risk-based and preference-based approaches at a population level**. *Cancer* 2014, **120**(19):2955-2964.

11. Karnon J, Brown J: **Selecting a decision model for economic evaluation: a case study and review**. *Health Care Manag Sci* 1998, **1**(2):133-140.

12. Karnon J: **Alternative decision modelling techniques for the evaluation of health care technologies: Markov processes versus discrete event simulation**. *Health Economics* 2003, **12**(10):837-848.

13. Albert U-S, Alt D, Kreienberg R, Naß-Griegoleit I, Schulte H, Wöckel A: **Patientenleitlinie "Brustkrebs: Früherkennung"**. In*.* Berlin: Leitlinienprogramm Onkologe; 2010.

14. NHS Cancer Screening Programmes: **Consolidated Guidance on Standards for the NHS Breast Screening Programme. Version 2**. In: *NHSBSP Publication.* vol. 60. Sheffield: NHS Cancer Screening Programmes; 2005.

15. U.S. Preventive Services Task Force: **Screening for Breast Cancer: U.S. Preventive Services Task Force Recommendation Statement**. *Ann Intern Med* 2009, **151**(10):716-726.

16. NABON: **Breast Cancer Guideline**. In*.* Edited by Zonderland HM, van Vegchel T. Amsterdam: Comprehensive Cancer Centre the Netherlands; 2012.

17. Yancik R, Wesley MN, Ries LA, Havlik RJ, Edwards BK, Yates JW: **Effect of Age and Comorbidity in Postmenopausal Breast Cancer Patients Aged 55 Years and Older**. *JAMA : the journal of the American Medical Association* 2001, **285**(7):885-892.

18. Jemal A, Ward E, Thun MJ: **Recent trends in breast cancer incidence rates by age and tumor characteristics among U.S. women**. *Breast Cancer Res* 2007, **9**(3):R28.

19. **Surveillance Epidemiology and End Results Program of the National Cancer Institute** [<http://seer.cancer.gov/seerstat>]

20. Brennan A, Chick SE, Davies R: **A taxonomy of model structures for economic evaluation of health technologies**. *Health Economics* 2006, **15**(12):1295-1310.

21. Schousboe JT, Kerlikowske K, Loh A, Cummings SR: **Personalizing Mammography by Breast Density and Other Risk Factors for Breast Cancer: Analysis of Health Benefits and Cost-Effectiveness**. *Ann Intern Med* 2011, **155**(1):10-20.

22. Stout NK, Lee SJ, Schechter CB, Kerlikowske K, Alagoz O, Berry D, Buist DSM, Cevik M, Chisholm G, de Koning HJ *et al*: **Benefits, Harms, and Costs for Breast Cancer Screening After US Implementation of Digital Mammography**. *JNCI Journal of the National Cancer Institute* 2014, **106**(6):dju092-dju092.

23. Sprague BL, Stout NK, Schechter C, van Ravesteyn NT, Cevik M, Alagoz O, Lee CI, van den Broek JJ, Miglioretti DL, Mandelblatt JS *et al*: **Benefits, harms, and cost-effectiveness of supplemental ultrasonography screening for women with dense breasts**. *Ann Intern Med* 2015, **162**(3):157-166.

24. Trentham-Dietz A, Kerlikowske K, Stout NK, Miglioretti DL, Schechter CB, Ergun MA, van den Broek JJ, Alagoz O, Sprague BL, van Ravesteyn NT *et al*: **Tailoring Breast Cancer Screening Intervals by Breast Density and Risk for Women Aged 50 Years or Older: Collaborative Modeling of Screening Outcomes**. *Ann Intern Med* 2016.

25. Tosteson AN, Stout NK, Fryback DG, Acharyya S, Herman BA, Hannah LG, Pisano ED, Investigators D: **Cost-effectiveness of digital mammography breast cancer screening**. *Ann Intern Med* 2008, **148**(1):1-10.

26. Vilaprinyo E, Forné C, Carles M, Sala M, Pla R, Castells X, Domingo L, Rue M, the Interval Cancer Study G: **Cost-Effectiveness and Harm-Benefit Analyses of Risk-Based Screening Strategies for Breast Cancer**. *PLoS ONE* 2014, **9**(2):e86858.

27. Ahern CH, Shih YCT, Dong W, Parmigiani G, Shen Y: **Cost-effectiveness of alternative strategies for integrating MRI into breast cancer screening for women at high risk**. *British Journal of Cancer* 2014:1-10.

28. Cott Chubiz JE, Lee JM, Gilmore ME, Kong CY, Lowry KP, Halpern EF, McMahon PM, Ryan PD, Gazelle GS: **Cost-effectiveness of alternating magnetic resonance imaging and digital mammography screening in BRCA1 and BRCA2 gene mutation carriers**. *Cancer* 2013, **119**(6):1266-1276.

29. Lee JM, McMahon PM, Kong CY, Kopans DB, Ryan PD, Ozanne EM, Halpern EF, Gazelle GS: **Cost-effectiveness of breast MR imaging and screen-film mammography for screening BRCA1 gene mutation carriers**. *Radiology* 2010, **254**(3):793-800.

30. Norman RP, Evans DG, Easton DF, Young KC: **The cost-utility of magnetic resonance imaging for breast cancer in BRCA1 mutation carriers aged 30-49**. *Eur J Health Econ* 2007, **8**(2):137-144.

31. Moore SG, Shenoy PJ, Fanucchi L, Tumeh JW, Flowers CR: **Cost-effectiveness of MRI compared to mammography for breast cancer screening in a high risk population**. *BMC Health Serv Res* 2009, **9**(1):9.

32. Pataky R, Armstrong L, Chia S, Coldman AJ, Kim-Sing C, McGillivray B, Scott J, Wilson CM, Peacock S: **Cost-effectiveness of MRI for breast cancer screening in BRCA1/2 mutation carriers**. *BMC Cancer* 2013, **13**(1):339.

33. Pataky R, Ismail Z, Coldman AJ, Elwood M, Gelmon K, Hedden L, Hislop G, Kan L, McCoy B, Olivotto IA *et al*: **Cost-effectiveness of annual versus biennial screening mammography for women with high mammographic breast density**. *Journal of Medical Screening* 2014, **21**(4):180-188.

34. Plevritis SK, Kurian AW, Sigal BM, Daniel BL, Ikeda DM, Stockdale FE, Garber AM: **Cost-effectiveness of screening BRCA1/2 mutation carriers with breast magnetic resonance imaging**. *JAMA : the journal of the American Medical Association* 2006, **295**(20):2374-2384.

35. Taneja C, Edelsberg J, Weycker D, Guo A, Oster G, Weinreb J: **Cost Effectiveness of Breast Cancer Screening With Contrast-Enhanced MRI in High-Risk Women**. *JACR Journal of the American College of Radiology* 2009, **6**(3):171-179.

36. Folse HJ, Green LE, Kress A, Allman R, Dinh TA: **Cost-effectiveness of a Genetic Test for Breast Cancer Risk**. *Cancer Prevention Research* 2013, **6**(12):1328-1336.

37. Manchanda R, Legood R, Burnell M, McGuire A, Raikou M, Loggenberg K, Wardle J, Sanderson S, Gessler S, Side L *et al*: **Cost-effectiveness of population screening for BRCA mutations in Ashkenazi jewish women compared with family history-based testing**. *Journal of the National Cancer Institute* 2015, **107**(1):380.

38. Ozanne EM, Esserman LJ: **Evaluation of breast cancer risk assessment techniques: a cost-effectiveness analysis**. *Cancer Epidemiol Biomarkers Prev* 2004, **13**(12):2043-2052.

39. Young JJ, Roffers S, Ries L, Fritz A: **SEER Summary Staging Manual - 2000: Codes and Coding Instructions**. In*.* Edited by Hurlbut A, vol. NIH Pub. No. 01-4969. Bethesda, MD: National Cancer Institute; 2001.

40. Lee SC, Jain PA, Jethwa SC, Tripathy D, Yamashita MW: **Radiologist's role in breast cancer staging: providing key information for clinicians**. *Radiographics* 2014, **34**(2):330-342.

41. Norman RP, Evans DG, Easton DF, Young KC: **The cost-utility of magnetic resonance imaging for breast cancer in BRCA1 mutation carriers aged 30-49**. *European Journal of Health Economics* 2007, **8**(2):137-144.

42. Fryback DG, Dasbach EJ, Klein R, Klein BE, Dorn N, Peterson K, Martin PA: **The Beaver Dam Health Outcomes Study: initial catalog of health-state quality factors**. *Medical decision making : an international journal of the Society for Medical Decision Making* 1993, **13**(2):89-102.

43. Lidgren M, Wilking N, Jönsson B, Rehnberg C: **Health related quality of life in different states of breast cancer**. *Qual Life Res* 2007, **16**(6):1073-1081.

44. Hanmer J, Lawrence WF, Anderson JP, Kaplan RM, Fryback DG: **Report of nationally representative values for the noninstitutionalized US adult population for 7 health-related quality-of-life scores**. *Medical decision making : an international journal of the Society for Medical Decision Making* 2006, **26**(4):391-400.

45. Stout NK, Rosenberg MA, Trentham-Dietz A, Smith MA, Robinson SM, Fryback DG: **Retrospective cost-effectiveness analysis of screening mammography**. *Journal of the National Cancer Institute* 2006, **98**(11):774-782.
